# The microbiota-gut- hippocampus axis

**DOI:** 10.3389/fnins.2022.1065995

**Published:** 2022-12-23

**Authors:** Mahmoud Salami, Masoud Soheili

**Affiliations:** Physiology Research Center, Institute for Basic Sciences, Kashan University of Medical Sciences, Kashan, Iran

**Keywords:** hippocampus, gut microbiota, learning and memory, synaptic plasticity, probiotics

## Abstract

**Introduction:**

It is well known that the intestinal bacteria substantially affect physiological processes in many body organs. Especially, through a bidirectional communication called as gut-microbiota-brain axis, the gut microbiota deeply influences development and function of the nervous system. Hippocampus, as a part of medial temporal lobe, is known to be involved in cognition, emotion, and anxiety. Growing evidence indicates that the hippocampus is a target of the gut microbiota. We used a broad search linking the hippocampus with the gut microbiota and probiotics.

**Methods:**

All experimental studies and clinical trials published until end of 2021 were reviewed. Influence of the gut microbiota on the behavioral, electrophysiological, biochemical and histological aspects of the hippocampus were evaluated in this review.

**Results:**

The effect of disrupted gut microbiota and probiotic supplements on the microbiota-hippocampus link is also considered. Studies show that a healthy gut microbiota is necessary for normal hippocampus dependent learning and memory and synaptic plasticity. The known current mechanisms are production and modulation of neurotrophins, neurotransmitters and receptors, regulation of intracellular molecular processes, normalizing the inflammatory/anti-inflammatory and oxidative/antioxidant factors, and histological stability of the hippocampus. Activity of the hippocampal neuronal circuits as well as behavioral functions of the hippocampus positively respond to different mixtures of probiotic bacteria.

**Discussion:**

Growing evidence from animal researches indicate a close association between the hippocampus with the gut microbiota and probiotic bacteria as well. However, human studies and clinical trials verifying such a link are scant. Since the most of papers on this topic have been published over the past 3 years, intensive future research awaits.

## 1 Introduction

The gut microbiota refers to all the species of commensal cells comprising approximately 100 trillion microbes that inhabits the entire gastrointestinal tract. With over 1,000 species and 7,000 strains the microbiota is known as a dynamic ecosystem dominated by anaerobic bacteria, but also includes viruses and bacteriophages, protozoa, archaea, and fungi (Lankelma et al., 2015). The two most prominent phyla of the gut microbiota present in human are Firmicutes and Bacteroidetes (Eckburg et al., 2005; Qin et al., 2010; Bäckhed, 2011). It encompasses almost 1–2 kg in adult human (Forsythe and Kunze, 2013; Toda et al., 2019) that equals weight (1.5 kg) of a normal adult brain (Parent and Carpenter, 1996). Although in primary studies the usefulness of the gut microbes was mostly considered in the gastrointestinal tract, however, it was found that, through production of a variety of bioactive substances, this intestinal flora impacts various body organs. Especially, the gut microbiota has a bidirectional communication with the nervous system called as the “microbiota-gut-brain axis” (Cryan and O’Mahony, 2011). This mutual connection occurs through several routs including neural, immune, metabolic and endocrine pathways (Novotny et al., 2019), and an intact gut microbiota is required for proper brain function (Grenham et al., 2011).

The brain is an organ that is more susceptible to both internal and external environmental factors in adolescence and early adulthood. During this period, the neuronal architecture and function undergo rapid modulation to cope with environmental challenges (Grenham et al., 2011). The gut microbiota plays a role in developmental programming of the brain, specifically synapse maturation, and synaptogenesis (Diaz Heijtz et al., 2011).

Hippocampus is the well-known area of brain involved in behavioral function. Particularly, it is a critical brain region associated with learning and memory and, in turn, is closely related to dementia and many other mental disorders such as depression and anxiety (Toda et al., 2019). For these, it is not surprising that the hippocampus has long term been under focus of behavioral and electrophysiological investigations. Histologically regular neuronal circuits of hippocampus have been subject of the experimental forms of synaptic plasticity, long term potentiation (LTP) and long term depression (LTD); both are known as candidate mechanisms of learning and memory.

Favorable effect of the gut microbiota on, at least, some cognitive brain function undoubtedly must be preceded through affecting the hippocampus. Although still scarce, however, evidence suggesting a direct link between the gut microbiota and hippocampal formation is increasing. In line, numerous studies have shown that the intestinal microbiota may affect hippocampal-related behaviors including learning and memory (Tang et al., 2021). The hippocampus appears to be particularly (Moloney et al., 2017) subject to the microbiota-gut-brain axis signaling related to neuronal morphology, neurogenesis, and neurotransmission (Clarke et al., 2013; Ogbonnaya et al., 2015; Luczynski et al., 2016).

Normal function of the brain strongly depends on natural composition of the gut flora, known as “Eubiosis” (Zhang et al., 2015). “Dysbiosis,” on the other hand, is reflected in decreased intestinal biodiversity or increased pathogenic bacteria. Under dysbiosis, the messages sent to the brain propagate unhealthy signals leading to inflammation, oxidative stress, unbalanced homeostasis of energy and increased cellular degeneration (Noble et al., 2017). Oxidative stress and neuroinflammation, in turn, are two major systemic conditions that aggravate neurodegeneration. The alterations occurred in the gut microbiota affect many aspects of the hippocampus through the vagus nerve pathway, the systemic pathway to increase the permeability of mucosa-intestinal barrier and blood-brain barrier and finally regulate hippocampus-dependent cognition and behaviors (Tang et al., 2021).

The composition and diversity of intestinal bacteria, the neuroactive substances, proinflammatory and inflammatory factors, and the levels of some gut microbiota metabolites such as lipopolysaccharide (LPS), trimethylamine N-oxide (TMAO) and amyloid beta (Aβ) in the gut microbiota could be affected by many treatments including antibiotics, germ free administration, diet, probiotics, prebiotics, and fecal microbiota transplantation (Tang et al., 2021). Probiotics, defined as live microorganisms with capability of promoting health to human and animal hosts when administered adequately and continuously (Sherman et al., 2009). The prominent commonly used probiotics to target the central nervous system (CNS) functions are mostly composed of two lactic acid producing bacteria genera including *Lactobacilli* and *Bifidobacteria* which are also normally found in healthy gut (Bodera and Chcialowski, 2009; Azad et al., 2018). Hence, it is not surprising that probiotics are considered to be used to repair damaged gut microbiota (Kwok et al., 2014). Probiotics influence dysfunction of the CNS in neurological disorders by increasing both diversity and count of the gut bacteria (Kwok et al., 2014). Evidence from animal research indicates that probiotic administration affects behavior, neurobiochemistry, and histology of hippocampus (Liu et al., 2015; Ho et al., 2019; Rahmati et al., 2019). This systematic review was mainly aimed to explore a link between the gut microbiota with behavioral, electrophysiological, biochemical and histological aspects of the hippocampus. The favorable role of probiotics on the above-mentioned aspects of hippocampus is also considered.

## 2 Methods

### 2.1 Search strategy

We used a broad search string including “hippocampus AND gut microbiota” or “hippocampus AND probiotics.” All papers published until end of 2021 were considered in Web of Science and PubMed. Additional papers were obtained from references of identified papers. Registries for clinical trials were checked.

### 2.2 Study selection

**FIGURE 1:**
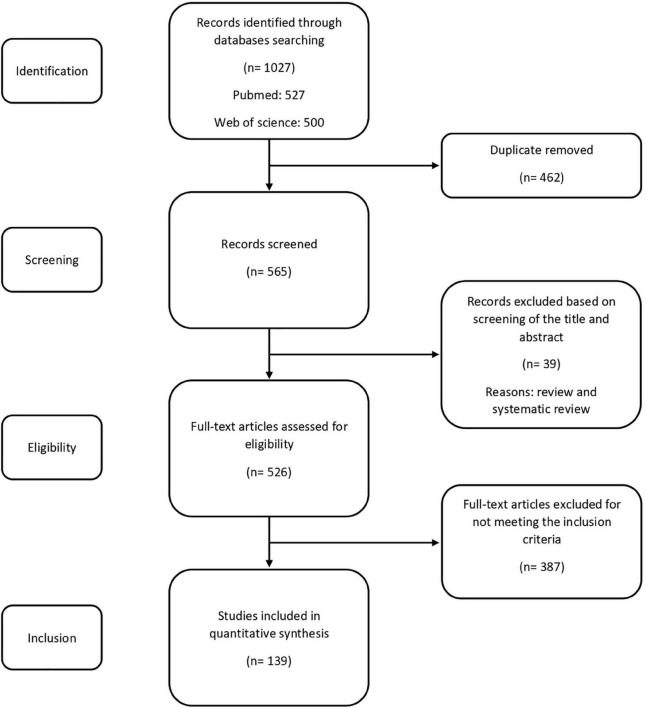
Flow diagram showing selection of the reviewed studies.

Preferred Reporting Items for Systematic Reviews and Meta-Analyses (PRISMA) course of action were used in this study (Figure 1). The main criteria for selecting papers were experimental studies that correlate the gut microbiota and/or probiotics to one of the following aspects: learning and memory, synaptic plasticity, LTP, neurotransmitter and neuromodulator alterations in the hippocampus, histological changes in the hippocampus, response of the hippocampus to changes in the plasma and cerebrospinal fluid levels of biochemical factors such as proinflammatory or inflammatory factors, cytokines, oxidants and antioxidants. Our database search yielded articles. All articles pertaining to the comprehensive, systematic and minireviews, non-original studies such as book reviews, letters to editor and editorials were excluded in this review; except the contents for preliminary studies and discussion, when necessary. The titles and abstracts of all articles were screened and selected further review if met the inclusion criteria. In total, full-text articles were assessed for eligibility and 139 met the above-mentioned criteria for review.

### 2.3 Quality assessment

First-grade studies were those that directly link hippocampus to the gut microbiota and probiotics. The second-grade studies were those working on the factors from the others organs (e.g., gastrointestinal tract) or other parts of the CNS that affect the hippocampus.

## 3 Results

The reviewed studies were those focused on relationship of the gut microbiota and probiotic with the hippocampus. The findings consider hippocampus-dependent cognitive function, level of brain derived nerve factor (BDNF) in the hippocampus, the hippocampus synaptic plasticity, the histological changes in the hippocampus and the balance of anti-oxidant/oxidant and anti-inflammatory/inflammatory factors. Table 1 briefly describe characteristics of the studies included in the systematic review. Also, details of the reviewed papers are given in the Supplementary Table 1.

**TABLE 1:** Brief description of studies indicating a link between the gut microbiota and the hippocampus.

Topic	Number of studies	Experimental model	Intervention	Findings
Learning and memory (12 studies)	(7 studies)	Probiotic treated animals	*Streptococcus thermophilus, Akkermansia muciniphila, Porphyromonas gingivalis, Streptococcus faecalis, Bacillus mesentericus*	Restored impaired learning and memory
	(1 studies)	Gut microbiota disrupted animals	Antibiotic therapy	Impaired learning and memory
	(3 studies)		Fecal microbiota transplantation From schizophrenic people, animals under high fat diet regimen and chronic unpredictable stress	Impaired learning and memory
	(1 study)		Fecal microbiota transplantation from aged people	Impaired learning and memory in recipients
Synaptic plasticity (15 studies)	(7 studies)	Probiotic treated animals	Different species of *Lactobacilli* and *Bifidobacteria, Streptococcus salivarius*	Enhanced LTP
	(2 studies)			Improved synaptic plasticity
	(1 study)			Decreased LTP
	(5 study)	Gut microbiota disrupted animals	Fecal microbiota transplantation from aged donor	Reduced expression of proteins involved in synaptic plasticity, reduces glutamatergic currents, dendritic signaling, intrinsic excitability of hippocampal neurons
			Dysbiosis	
BDNF level in hippocampus (27 studies)	(20 studies)	Probiotic treated animals	Different species of *Lactobacilli* and *Bifidobacteria, Streptococcus thermophilus*, *Enterococcus faecium, Clostridium butyricum, Streptococcus salivarius*	Increased the level of BDNF in the hippocampus
	(2 studies)			No change in BDNF level
	(1 study)	Gut microbiota disrupted animals	Fecal microbiota transplantation From aged donors	Reduced hippocampal expression of BDNF
	(4 studies)		Antibiotic therapy	Decreased hippocampal BDNF
Inflammatory/anti-inflammatory balance (37 studies)	(2 studies)	Probiotic treated animals	Different species of *Lactobacilli* and *Bifidobacteria, Akkermansia muciniphila, Porphyromonas gingivalis, Mycobacterium vaccae, Streptococcus thermophiles, Agathobaculum butyriciproducens, Escherichia coli*	Attenuate inflammation
	(1 study)			Reduces expression of inflammatory cytokines
	(1 study)			Decreases microglial activation- induced inflammation
	(4 studies)			Suppresses NF-κB activation
	(10 studies)			reduces TNF-α expression
	(5 studies)			Decreases expression of the proinflammatory interleukin IL- 1β
	(5 studies)			Decreases expression of the proinflammatory interleukin I L- 6
	(1 studies)			Increased the anti-inflammatory interleukin IL-10
	(1 study)	Gut microbiota disrupted animals	Gut microbiota metabolite, TMAO	Increases proinflammatory cytokine expression
	(1 study)			Increases microglia-mediated neuroinflammation
	(1 study)		Antibiotic therapy	Decreased proinflammatory cytokines IFN-γ and IL-17A levels and increased anti-inflammatory cytokine IL-10 and Increased hippocampal TNF-α and IL-1β
	(1 study)			Increased the recruitment of microglia and monocytes to the hippocampus and induced NF-κB activation and IL-1β, IL-6 and TNF-α expression in the brain
	(1 study)		Fecal microbiota transplantation from chronic mild stress treated animals	Increased IL-6 and TNF-α
	(1 study)		Fecal microbiota transplantation From aged subjects	Increased levels of pro- inflammatory cytokines in hippocampus
	(1 study)		Fecal microbiota transplantation from AD patients	Increased levels of inflammatory factors in both peripheral blood and the hippocampus
	(1 study)		Germ free animals	Enhanced levels of IL-2, IL-4 IL-6, IL-10, IL-17A and TNF-α in hippocampus and decreased IL- 4
Oxidant and anti-oxidant factors (18 studies)	(1 studies)	Probiotic treated animals	Different species of *Lactobacilli* and *Bifidobacteria, laccoccus, Clostridium butyricum, Enterococcus faecium, Streptococcus faecalis, Bacillus mesentericus*	Reduced hippocampal oxidative stress
	(5 studies)			Attenuated oxidative enzymes
	4 studies)			Increases antioxidant activity
	(4 studies)			Increased antioxidant/oxidant ratio
	(1 studies)	Gut microbiota disrupted animals	Gut microbiota metabolite, TMAO	Promote oxidative stress in the hippocampus
	(2 studies)			Decreased antioxidant activities in the hippocampus
	(1 studies)		From Fecal Microbiota transplantation	Increased level of oxidative stress
Pathological changes (3 studies)	(4 studies)	Probiotic treated animals	Different species of *Lactobacilli, Clostridium butyricum*	Attenuated the histopathological changes in the hippocampus
Apoptosis (9 studies)	(2 studies)	Probiotic treated animals	Different species of *Lactobacilli* and *Bifidobacteria, Streptococcus Thermophilus, Lactococcus lactis, Clostridium butyricum*	Decreased concentrations of the apoptotic agents Bax and cleaved caspase-3
	(3 studies)			Increased expression of anti- apoptotic genes (*Bcl-2*) in the hippocampus
	(3 studies)			Suppressing hippocampal apoptosis
	(1 studies)	Gut microbiota disrupted animals	Antibiotic therapy	Increased population of apoptotic neuron cells
Amyloid beta plaque (8 studies)	(8 studies)	Probiotic treated animals	Different species of *Lactobacilli* and *Bifidobacteria*	Reduces deposition of Aβ in the hippocampus

BDNF, brain derived neurotrophic factor; LTP, long term potentiation; TMAO, trimethylamine N-oxide.

### 3.1 Learning and memory

Twelve studies have explored a link between hippocampus-dependent learning and memory with the disrupted gut microbiota and the helpful role of probiotic supplements. Results of one study showed that damage to the gut microbiota leads to impaired learning and memory (Zeraati et al., 2019). Seven studies have considered the effect of probiotics on hippocampus-dependent learning and memory in the animal models. *Lactobacilli* and *Bifidobacteria* were the most probiotic bacteria used in these studies. *Streptococcus thermophilus*, *Akkermansia muciniphila*, *Porphyromonas gingivalis*, *Streptococcus faecalis*, and *Bacillus mesentericus* were the other administered probiotics. In all cases the probiotic therapy improved the impaired learning and memory (Zhang et al., 2018; Ho et al., 2019; McVey Neufeld et al., 2019; Rahmati et al., 2019; Liu et al., 2020; Roy Sarkar et al., 2020; Cui et al., 2021; Zolfaghari et al., 2021). Three studies reported that fecal microbiota transplantation from schizophrenic people (Zhu et al., 2020), high fat diet (Yang et al., 2019) and chronic unpredictable stress (Shan et al., 2020) gave rise to impaired learning and memory. One study indicated that TMAO may affect social behaviors by regulating metabolites in the hippocampus (Luo et al., 2021). One study confirmed positive role of gut microbial metabolite short-chain fatty acids (SCFAs) in cognitive deficits (Xu et al., 2021). In one clinical trial, fecal microbiota transplantation from aged donors weaken spatial learning and memory in young adult recipients (D’Amato et al., 2020).

### 3.2 Synaptic plasticity

Hippocampal synaptic plasticity following probiotic therapy and fecal microbiota transplantation was evaluated in 15 studies. Different species of *Lactobacilli* and *Bifidobacteria*, and *Streptococcus salivarius* were used as probiotic bacteria. The probiotic administration enhanced LTP in 6 studies (Davari et al., 2013; Distrutti et al., 2014; Asl et al., 2019; Rezaei Asl et al., 2019; Rezaeiasl et al., 2019; Talani et al., 2020). Two studies reported improving effect of probiotics on the synaptic plasticity (Luck et al., 2020; Wang et al., 2020a). One study showed striking alteration of hippocampal LTP in male germ-free slices (Darch et al., 2021). On the other hand, one study reported decreased hippocampal LTP following probiotic administration (Tahmasebi et al., 2020). Fecal microbiota transplantation from aged donor mice reduced expression of proteins involved in synaptic plasticity (D’Amato et al., 2020).

Evidence indicates that while dysbiosis alters neuronal functions, reduces spontaneous postsynaptic glutamatergic currents (Cordella et al., 2021), alters integration of dendritic signaling (Darch et al., 2021) and decreases intrinsic excitability in hippocampal neurons (Kim et al., 2022) probiotic treatment selectively increases the intrinsic excitability of hippocampal CA1 pyramidal neurons (Kim et al., 2022).

### 3.3 BDNF level in the hippocampus

Twenty-seven studies investigated relevancy of the BDNF concentration in the hippocampus with the altered gut flora and the probiotic treatments. The main administered probiotics were *Lactobacilli* and *Bifidobacteria*; however, other probiotics such as *Streptococcus thermophilus*, *Enterococcus faecium*, *Clostridium butyricum*, *Streptococcus salivarius*, and *Prevotella histicola* were also used. In most of the studies the probiotic treatment increased the level of BDNF in the hippocampus (Distrutti et al., 2014; Liang et al., 2015; Liu et al., 2015; Corpuz et al., 2018; Jang et al., 2018a,2019a,2019b; Romo-Araiza et al., 2018; Lee et al., 2019; Sovijit et al., 2019; Tsukahara et al., 2019; Han et al., 2020a,b; Kim et al., 2020; Mohammed et al., 2020; Talani et al., 2020; Yang et al., 2020; Yun et al., 2020; Zhang et al., 2020). However, in two studies the probiotic treatments could not change in the level of BDNF (O’Sullivan et al., 2011; Rahmati et al., 2019). The probiotic treatment also elevated the BDNF expression in cultured hippocampal neurons (Choi et al., 2019). Fecal microbiota transplantation from the aged donors reduced the hippocampal expression of BDNF in one study (Li et al., 2020). Using a mixture of antibiotics, four studies induced gut microbiota depletion. The results showed that hippocampal BDNF was significantly decreased in these animals (Olson et al., 2018; Bistoletti et al., 2019; Zeraati et al., 2019; Lee et al., 2020).

### 3.4 Histopathological considerations

The effect of probiotic supplementations on histopathological changes of the hippocampus was assessed in animal models. *Lactobacilli* species and *Clostridium butyricum* were used as probiotics. Results showed that the probiotic treatments attenuated the histopathological changes in the hippocampus (Liu et al., 2015; Sun et al., 2016; Mohammed et al., 2020).

### 3.5 Apoptosis

Nine studies have focused on apoptosis. Beside the main bacteria *Lactobacilli* and *Bifidobacteria*, the other administered probiotic bacteria were *Streptococcus thermophilus*, *Lactococcus lactis*, and *Clostridium butyricum*. All studies reported positive effect of the probiotics in prevention of apoptosis in the hippocampus. Two studies reported decreased concentrations of the apoptotic agents Bax and cleaved caspase-3 (Sun et al., 2016; Xu et al., 2018). Also, 3 studies showed increased expression of anti-apoptotic genes (*Bcl-2*) in the hippocampus (Liu et al., 2015; Sun et al., 2016; Xin et al., 2020). The results of two studies showed prevention of the hippocampal apoptosis (Mohammadi et al., 2019; Rahmati et al., 2019). One study reported the protective effects of probiotics on mitochondrion-mediated apoptosis in the hippocampus (Wang et al., 2020a). Conversely, fecal microbiota transplantation from the antibiotic-induced microbiota disrupted mice resulted resulted in increased population of apoptotic neuron cells (Jang et al., 2018a,b).

### 3.6 Amyloid beta plaques

Eight studies assessed if probiotic supplementation underlies Aβ deposition in the hippocampus. Different species of *Lactobacilli* and *Bifidobacteria* were used as probiotics. All studies found that the probiotic treatment reduces deposition of Aβ in the hippocampus (Athari Nik Azm et al., 2018; Abraham et al., 2019; Lee et al., 2019; Liu et al., 2020; Mehrabadi and Sadr, 2020; Wang et al., 2020b; Wu et al., 2020; Go et al., 2021).

### 3.7 Oxidant and anti-oxidant factors

Eighteen studies have searched the effect of probiotic therapy on the oxidants/antioxidants factors in the hippocampus. The administered probiotics were *Lactobacilli*, *Bifidobacteria*, *Lactococcus lactis*, *Clostridium butyricum*, *Enterococcus faecium*, *Streptococcus faecalis*, and *Bacillus mesentericus*. One study showed that probiotic treatment reduced the hippocampal oxidative stress (Chunchai et al., 2018). Five studies reported that probiotic supplementations attenuated oxidative enzymes (Sun et al., 2016; Alchujyan et al., 2018; Xu et al., 2018; Ramalho et al., 2019; Salami et al., 2019). Consistently, 4 studies showed that probiotic administration increases antioxidant activity (Divyashri et al., 2015; Huang et al., 2018; Ho et al., 2019; Roy Sarkar et al., 2020). Four studies reported an increased antioxidant/oxidant ratio in the probiotic treated animals (Sun et al., 2016; Xu et al., 2018; Rezaeiasl et al., 2019; Mehrabadi and Sadr, 2020). Gut microbiota-derived metabolite TMAO promotes oxidative stress and downregulates the antioxidant methionine sulfoxide reductase A (MsrA) enzyme in the hippocampus, which may sensitize the hippocampus to oxidative stress. In two studies TMAO decreased antioxidant activities in the hippocampus (Meng et al., 2019; Zhao et al., 2019). In one study age-related fecal microbiota transplantation increased level of oxidative stress in young rats (Li et al., 2020). In a study fecal microbiota transplantation elevated SOD and CAT activities and GSH/GSSG ratio and diminished ROS, GSSG, and MDA levels in the hippocampus after traumatic brain injury (Du et al., 2021).

### 3.8 Inflammatory and anti-inflammatory factors

Fifty-three researches found a relation between gut microbiota and probiotics with Inflammatory and anti-inflammatory factors in the hippocampus. Of them, thirty-seven studies tested inflammatory responses of hippocampus to probiotic administration. Again, *Lactobacilli* and *Bifidobacteria* were the main administered probiotics and the other bacteria were *Akkermansia muciniphila*, *Porphyromonas gingivalis*, *Mycobacterium vaccae*, *Streptococcus thermophiles*, *Agathobaculum butyriciproducens*, *Escherichia coli*, and *Prevotella histicola*.

Probiotic administrations are reported to attenuate inflammation (Ni et al., 2019; Wang et al., 2020b), reduce expression of inflammatory cytokines (Guo et al., 2019) and decreases microglial activation-induced inflammation (Chunchai et al., 2018). Evidence indicates that probiotics increase anti-inflammatory cytokines and reduce inflammatory cytokines. Probiotic administration suppresses NF-κB activation (Jeong et al., 2016; Olson et al., 2018; Lee et al., 2019; Han et al., 2020a). Also, probiotic therapy reduces the expression of proinflammatory cytokine TNF-α (Sousa et al., 2008; Jeong et al., 2016; Lee et al., 2019; Lin et al., 2019; Yang et al., 2019; Han et al., 2020a,b; Mehrabadi and Sadr, 2020; Wu et al., 2020; Zolfaghari et al., 2021). Probiotics also decrease expression of the proinflammatory IL-1β (Jeong et al., 2016; Lin et al., 2019; Yang et al., 2019; Mehrabadi and Sadr, 2020; Wu et al., 2020), IL-6 (Abildgaard et al., 2017; Yang et al., 2019; Han et al., 2020a,b; Wu et al., 2020; Xiao et al., 2020) and IFN-γ (Ma et al., 2021). On the other hand, it is reported that probiotic supplementation increased the anti-inflammatory IL-10 (Wang et al., 2020a).

**FIGURE 2:**
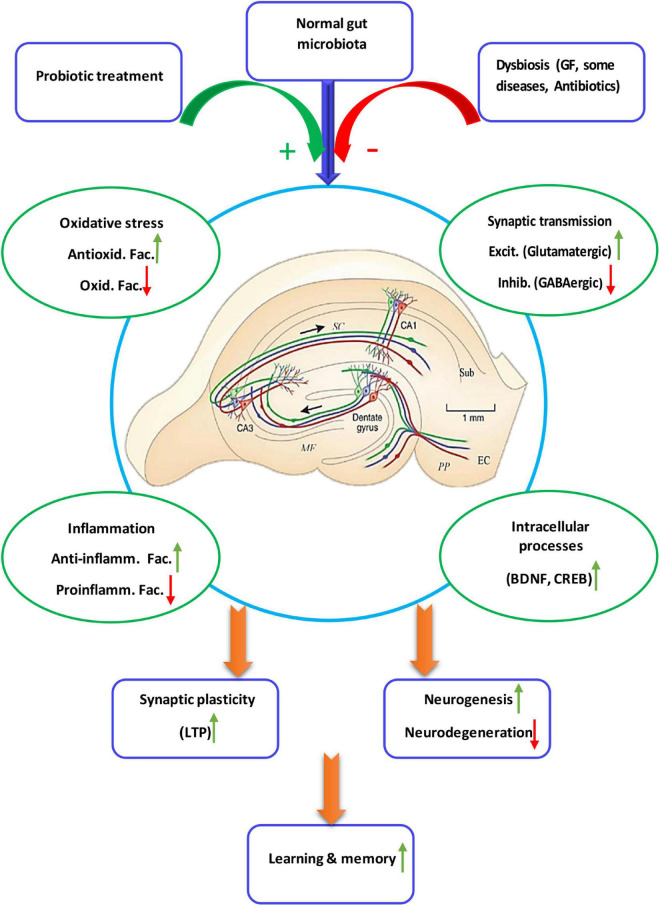
Schematic diagram showing details of the microbiota-gut-hippocampus axis at different levels. The normal gut microbiota enhances synaptic plasticity and, thus, hippocampal-dependent learning and memory, through a balance of anti-inflammatory/inflammatory and antioxidant/oxidant agents, stimulatory/inhibitory synaptic transmission, as well as enhancement of intracellular devices. While intestinal microbiota negatively affect biochemistry, immunology, neurochemistry and intracellular cascades of synaptic plasticity and cognition, probiotic treatment restores dysbiotic hippocampal disorders. Anti-inflamm. Fac., anti-inflammatory factors; Proinflamm. Fac., proinflammatory factors; Antioxid. Fac., antioxidant factors, Oxid. Fac., oxidant factors; Excit, excitatory, Inhib., inhibitory.

Two studies proved that TMAO increases expression of proinflammatory cytokines (Zhao et al., 2019) and reactive oxygen species (ROS) microglia-mediated neuroinflammation (Meng et al., 2019). One study showed that antibiotic-induced gut microbial dysbiosis after traumatic brain injury increased neuroinflammation (Celorrio et al., 2021). In another study it was found that antibiotic-induced microbiota depletion decreases the proinflammatory cytokines IFN-γ and IL-17A levels and increases the anti-inflammatory cytokine IL-10. It also increased hippocampal TNF-α and IL-1β in EAE-induced mice (Zeraati et al., 2019). Four studies applied the fecal microbiota transplantation technique. In one study fecal microbiota transplantation of mice treated with ampicillin increased the recruitment of microglia and monocytes to the hippocampus. It also induced NF-κB activation and expression of IL-1β, IL-6, and TNF-α in the brain (Jang et al., 2018a). Another study showed that fecal microbiota transplantation from chronic mild stress treated animals increased IL-6 and TNF-α (Marcondes Ávila et al., 2020). Age-related fecal microbiota transplantation increased levels of pro-inflammatory cytokines in hippocampus (Li et al., 2020). Fecal microbiota transplantation from the patients with Alzheimer’s disease increased levels of inflammatory factors in both peripheral blood and the hippocampus (Shen et al., 2020). Studies on the germ free animals, the animals deprived from natural gut flora, showed enhanced levels of IL-2, IL-4, IL-6, IL-10, IL-17A, and TNF-α (Olson et al., 2018; de Miranda et al., 2020) and decreased level of IL-4 (Wu et al., 2020) in the hippocampus. Figure 2 illustrate the relationship of the gut microbiota, under eubiosis and dysbiosis conditions, and probiotic bacteria with the hippocampal related cognitive functions and synaptic plasticity.

## 4 Discussion

Majority of the reviewed papers studying either the normal or disrupted gut microbiota indicates a strong link between the hippocampus-dependent learning and memory and synaptic plasticity with the intestinal flora. Growing proofs indicating considerable role of beneficial bacteria from either the gut microbiota or probiotics in the axis mainly comes from the studies carried out in the disrupted gut microbiota and germ free subjects. It has been shown that the hippocampus-related behaviors such as anxiety, depression, and cognitive function are abnormal in germ free animals. Diaz Heijtz et al. (2011), found that 50 genes in the hippocampus of germ free animals are significantly different (higher or lower) expressed in compared with those with normal gut microbiota. On the other hand, supporting the damaged gut microbiota by probiotic supplements restores its normal function. Also, fecal microbiota transplantation technique confirms necessity of a healthy gut microbiota for normal hippocampal dependent phenomena.

The hippocampal dependent cognitive function is mechanistically contributed to occurrence of an important form of synaptic plasticity, LTP. The hippocampal LTP is essential for the coding and storage of long term spatial memories so that prevention of LTP induction in the hippocampus affects the spatial learning (Bannerman et al., 2014). Therefore, having a cross talk between the LTP and the learning and memory, all factors affecting the LTP deeply influence the cognitive behaviors. A key regulator of the LTP itself is the neurotrophin BDNF (Monteggia et al., 2004). The BDNF is known to be associated with generating and preserving LTP in the CA1 region of the hippocampus (Diógenes et al., 2011; Katche et al., 2013) and therefore it plays an important role in the cognitive function (Pang and Lu, 2004). The cognitive impairments are reported to be partly mediated by altered BDNF expression in the hippocampus (Frohlich et al., 2016). Except a few, most of the reviewed studies demonstrated that the gut microbiota disruption reduced the hippocampal expression of BDNF. Literature reviewed here shows that probiotic treatment strengthens the suppressed LTP in the CA3-CA1 pathway of the hippocampus. Therefore, modulation of the BDNF by the gut microbiota and probiotic bacteria partly explains its role in regulation of synaptic transmission and, thus, cognitive function of the hippocampus.

The LTP in the CA3-CA1 pathway of the hippocampus is NMDA receptor-dependent. Altered expression of some neurotransmitters or their receptors is observed in the animals with absent or disrupted gut microbiota. The NMDA receptor level in the hippocampus is decreased in the damaged gut microbiota (Wang et al., 2015) and the germ free animals (Neufeld et al., 2011). Microbiome fecal transplants from patients with schizophrenia heads to lower glutamate and higher glutamine and GABA in the hippocampus (Zheng et al., 2019). Bravo et al. observed enhanced memory consolidation, with decreased hippocampal GABAB1b mRNA; suggesting a potential mechanism by which cognitive behavior may be modulated (Athari Nik Azm et al., 2018). Also, absence of gut microbiota significantly upregulates the glucocorticoid receptor pathway genes in hippocampus (Cristiano et al., 2018). From these considerations it can be concluded that a mechanism by which the helpful bacteria may affect the hippocampus-dependent cognitive function is influencing neurotrophins, neurotransmitters, and receptors.

Unexpected to many other areas of brain, neurogenesis occurs in the adult hippocampus and it is believed that this process importantly influences learning and memory (Marín-Burgin and Schinder, 2012). It is reported that there is a connection between the gut microbiota composition and their metabolic function to neurogenesis in the adult hippocampus (Wei et al., 2021) so that antibiotic-induced gut microbial dysbiosis can increase hippocampal neuronal loss (Celorrio et al., 2021).

It is reported that a path by which microbiota is able to affect the CNS functions is the change of hippocampal neurogenesis in adults (Kempermann et al., 2015). Consistently, numerous findings suggest that some brain processes which are regulated by neurogenesis in adult hippocampus are affected in germ free animals (Gareau et al., 2011). These evidences indicate that, through histological support, the gut microbiota can underlie the hippocampal function.

Another mechanism by which the gut microbiota impacts the hippocampus is its important role in modulating microRNAs (miRNAs); the small, non-coding RNAs which emerge as key post-transcriptional modulators of gene expression (Cui et al., 2014). They influence hippocampal gene expression (Moloney et al., 2017) and, thus, considerably affect the processes critical to hippocampal development (Moloney et al., 2017). Inhibition of miRNAs in the hippocampus results in changes in cognitive behavior in rodents (Malmevik et al., 2016) and it is suggested that many of the behavioral effects mediated by the gut microbiota are controlled by miRNAs (Moloney et al., 2017). Studies also show that the cyclic-adenosine-monophosphate response element binding (CREB) in hippocampal neurons is regulated by the gut microbiota. It is shown that absence of the gut microbiota from birth is associated with decreased hippocampal CREB (Zeng et al., 2016). The CREB regulates the genes related to neuronal differentiation, synaptic plasticity, and learning and memory (Murray et al., 2019). Therefore, gene expression could be an intracellular path through which the gut microbiota regulates hippocampus-related synaptic plasticity and cognition.

This review demonstrates that the intestinal bacteria has a powerful influence on the neuroinflammation so as to the gut microbiota has been proved to be an important neuroimmunomodulator (Foster, 2016; Rea et al., 2016). Evidence indicate that any damage to the gut microbiota increases the inflammatory factors in the hippocampus. It is demonstrated that there is a specific inflammatory pattern, in dorsal and ventral hippocampus, that is significantly correlated with gut microbiota composition (Lopizzo et al., 2021). Gut dysbiosis is shown to contribute to both peripheral and central inflammatory processes and cognitive deficits (Wang et al., 2021).

Neuroinflammation is associated with the cognitive impairment and memory decline, as the hippocampus is prone to develop alterations in synaptic transmission and plasticity during inflammation (Di Filippo et al., 2013). Whereas secretion of pro-inflammatory cytokines may be inhibited by BDNF, neuroinflammation, itself, is shown to negatively affect the hippocampal expression of BDNF (Mora, 2013; Moreno-Fernández et al., 2013; Ryan and Nolan, 2016). One of the biological changes related to inflammation is the activity of cytokines; the proteins that regulate inflammation (Scheller et al., 2011). Cytokines can modulate the neurogenesis, the synapse formation and plasticity (Hodes et al., 2015), influence the activity of neurons, astrocytes and microglia (see below) in the CNS (Kohman and Rhodes, 2013) and, thus, impact the cognition and mood (Valkanova et al., 2013; Khandaker et al., 2014). Especially, the microglia are the resident immune cells in the brain and has been causally linked to developmental neuronal cell death (Marín-Teva et al., 2011). These immune cells respond to perturbations by initiating an immune response involving the release of proinflammatory cytokines and actively promote developmental cell death in the hippocampus and cerebellum (Wakselman et al., 2008; Marín-Teva et al., 2011). On the other hand, the papers reviewed here revealed that probiotic administrations reduce inflammation through attenuating expression of the inflammatory cytokines and the microglial induced inflammation. Further, the probiotics positively affect production of the anti-inflammatory factors. Also, evidence from oxidative and antioxidative activities proves that the probiotic bacteria increases the antioxidative activities in cost of suppressing the oxidative activities. Conversely, some gut microbiota-derived metabolites (discussed below) as well as fecal microbiota transplantation from aged people proceed the oxidative stress in the hippocampus of recipient subjects. Overall, the gut and probiotic bacteria auspiciously balance inflammation and oxidation; in favor of anti-inflammatory and antioxidant agents.

The intestinal microbiota is responsible for a significant production of SCFA (Romo-Araiza et al., 2018) and the probiotics are known to highly influence production of the SCFAs. Production of butyrate, the most important SCFA, by healthy gut microbiota can facilitate LTP and the formation of long term memory (Levenson et al., 2004). It is reported that the probiotics can improve learning and memory through an increase in the butyrate that, in turn, increases BDNF and decreases the proinflammatory cytokine concentrations in the hippocampus (Romo-Araiza et al., 2018). In addition, the expression of enzymes involved in the production of antioxidant enzymes can be triggered by butyrate production (Hamer et al., 2009; Aguilar et al., 2016; Bourassa et al., 2016; Slattery et al., 2016). The other SCFA propionate is known to up-regulate the protein CREB that, in turn, regulates synaptic plasticity, memory formation, and the reward system (Slattery et al., 2016).

Despite aforementioned valuable roles of the gut microbiota, it may play injurious role under adverse conditions of the gut flora. TMAO, a gut microbiota metabolite, increases proinflammatory cytokine expression and microglia-mediated neuroinflammation. Also, structural bacterial components such as LPS provide low grade tonic stimulation of the innate immune system. Higher abundance of LPS is detectable in hippocampus of the people with Alzheimer’s disease (Zhao et al., 2017). Dysfunctional responses of the adaptive immune system to some bacterial proteins and production of some neurotoxic metabolites such as D-lactic acid and ammonia are the other examples. Even helpful metabolites such as SCFAs may have neurotoxic effects (Galland, 2014).

## 5 Future implications and limitations

Current research shows a close relationship between several brain disorders and changes in the gut microbiota (Salami, 2021); promising favorable role of bacteria in diagnosis and/or treatment of the brain diseases. In particular, from a therapeutic point of view, it can be hypothesized whether fecal microbiota transplantation or the administration of probiotics can improve hippocampal-dependent learning and memory.

Because this systematic review was devoted to the studies directly related to the hippocampus, very little data was available from human research and clinical trials, and so, at least in terms of cognitive function, we are just getting started.

Therefore, human studies are encouraged to investigate the potential effects of the gut microbiota and probiotics on the hippocampus-related behaviors, particularly in brain diseases associated with cognitive disorders. Since majority of the papers considering relevancy of the hippocampus with the gut and probiotic bacteria have been published in the last 3 years, it promises a future with intensive research in this area.

## 6 Conclusion

Overall, the production and modulation of neurotrophins, neurotransmitters and receptors, regulation of intracellular molecular processes, balance of inflammatory/anti-inflammatory and oxidative/antioxidant factors, and preservation of structural aspects of the hippocampus can be considered as important ways through them the gut microbiota affect the synaptic activity in the hippocampal neural circuits and hippocampus related behaviors. The supportive role of the probiotic bacteria, whether as supplementation for health or as therapeutic tool, is appreciated. However, the degree of efficacy of probiotics, optimal application paths, sex specificity, ages with the best most vulnerability to probiotics and the duration of treatment should be considered.

## Data availability statement

The original contributions presented in this study are included in the article/Supplementary material, further inquiries can be directed to the corresponding author.

## Author contributions

MSa was the project leader of the review, designed the review, tabulated the review results, interpreted and discussed review findings, and did the screening, data abstraction, and quality appraisal. MSo conceived the review and prepared the tables and drawings. Both authors managed the review processes, summarized and described the review findings, and prepared the final version.
