# Supplementary material for: The microbiota-gut- hippocampus axis
Source: Front Neurosci. 2022 Dec 23;16:1065995. doi: 10.3389/fnins.2022.1065995 (PMC9817109; doi:10.3389/fnins.2022.1065995)
Supplement: Supplementary file 1 [file Table_1.doc]

**Suplementary table** - Characteristics of studies included in the systematic review

| Publication | Results | test | Treatment | Type of administration | Type of probiotics | Type of study/ sample size | Aim of study |
| --- | --- | --- | --- | --- | --- | --- | --- |
| Zhao et al. 2019 | - freezing to contextual fear conditionin - microglia activity - proinflammatory cytokine expression - NADPH oxidase-dependent ROS production in hippocampus - Hippocampal expression of antioxidant enzyme methionine sulfoxide reductase A | -Fear Conditioning Test  -Open Field Test  -Western Blot Analysis  -NADPH Oxidase Activity Assay  -Hydrogen Peroxide Assay  -Immunofluorescent Study  -Circulating TMAO and Proinflammatory Cytokine  Measurements | 3 weeks | in drinking water | trimethylamine N-oxide (TMAO) | Sprague–Dawley rats/60 | Effect of Trimethylamine N‑oxide on cognitive dysfunction |
| Salami et al. 2019 | - LTP - Amyloid beta plaque - TAC - MDA - total amount of microbiota | -Morris water maze  -Electrophysiological recording  -Anti-oxidant/oxidant factors assay  -Brain staining  -Fecal bacteria quantification | 8 weeks | Dissolve in drinking water | L. acidophilus  B. bifidum  B. longum | animal model of Alzheimer's disease (rats)/- | Effect of Probiotic on spatial cognitive performance  and synaptic plasticity |
| Ni et al 2019 | - oxidative stress - inflammation - learning and memory ability - neurodegenerative and neurotrophic factors expressions in hippocampus | -Grip strength test  -Forced swimming test  -Morris water maze Test  -New object recognition test  -Active shuttle avoidance test  -Histopathological analyses  -Quantitative RT-PCR analysis  -16S rRNA sequencing  -Data analysis of fecal contents | 12 weeks | oral | L. casei LC122 or B. longum BL986 | C57BL-6 mice/46 | Effect of probiotics on Physiological Function and Cognitive Ability |
| Lee et al. 2019 | - cognitive - NF-κB activation - TNF-α expression - Iba1^+^, LPS^+^/CD11b^+^, and caspase-3^+^/NeuN^+^ cell populations - NF-κB activation in the hippocampus - β/γ-secretases - caspase-3 expression - amyloid-β accumulation in the hippocampus | -Y-maze  -Immunofluorescence assay -Immunoblotting-Myeloperoxidase activity assay-Determination of LPS-Culture of fecal bacteria-Pyrosequencing | for 1 and 2 months in aged and Tg mice | oral | B. longum | 5XFAD  transgenic and aged mice/36 | Effect of commensal gut bacteria on cognitive decline in Alzheimer's disease |
| Zhu et al 2019 | - behavioral abnormalities - learning and memory | -Open field  -Reciprocal social interaction test  -Three chamber  social test  -Novel object test  -Forced  swimming test  -Elevated plus maze  -Barnes maze  Tail suspension test  -16S rRNA amplicon sequencing  -ELISA | 3 weeks | oral gavage | SCZ microbiota transplantation | SCZ patients and C57BL/6J mice/- | Effect of microbiota transplantation from drug-free patients with  schizophrenia on abnormal behaviors and  dysregulated kynurenine metabolism |
| Li et al. 2019 | - depressive-like behaviors - 5-HT in hippocampus. | -Sucrose preference test  -Measurement and analysis of central and colonic serotonin levels and relative metabolism factors  -Forced swimming test  -16S rRNA gene pyrosequencing | 4 weeks | oral | B. longum and L. rhamnosus | Wistar rat/50 | Effects of prebiotics and probiotics on the serotonin metabolism in the chronic unpredictable mild stress |
| [Yue](https://www.ncbi.nlm.nih.gov/pubmed/?term=Yue T%5BAuthor%5D&cauthor=true&cauthor_uid=31315772) et al 2019 | - degeneration and necrosis in hippocampus - in abundance and diversity of intestinal flora - in abundance of Proteobacteria at the phylum level - in abundance of Prevotella and Lactobacillus - in abundance of Staphylococcace and Oligella | -Morris water maze  -Hematoxylin and eosin staining  -16S rRNA sequencing  -principal component analysis (PCA) | 2 days | intraperitoneally | LPS | Sprague-Dawley rats/- | Structural features of intestinal flora in preterm rats with cognitive impairment |
| Rahmati et al. | - in number of apoptotic cells - neuronal death in the CA1, CA3 and DG of the hippocampus - spatial memory impairment - neurological dysfunction - hippocampus damage and - spatial learning and memory deficit | -Radial arm water maze -TUNEL assay-Cresyl violet staining-Measurement of the hippocampus MDA-FRAP content-BDNF level | 3 weeks | gastric gavages | L. casei  L. acidophilus  L. rhamnosus  L. bulgaricus  B. breve  B. longum  S. thermophilus | Swiss albino mice/50 | Effect of probiotic supplementation on hippocampus injury and spatial learning and memory impairments |
| Guo et al. 2019 | time spent in the center of the open field apparatuspercentage of entries into the open arms of the elevated plus-mazepercentage of time spent in the open arms of the EPMimmobility duration in the tail suspension test as well as the forced swimming testsequence proportion of Lactobacillussequence proportion of Bacteroides in fecesprotein expression of inflammatory cytokinesbrain derived neurotrophic factor (BDNF) expression in hippocampus | -Open Field Test-Elevated Plus-Maze-Tail Suspension Test-Forced Swimming Test-Analysis of fecal microflora-Western blotting of the hippocampus-Immunofluorescence labeling in the hippocampus | 3 weeks |  | B. adolescentis | ICR mice/86 | Effect of B. adolescentis on anxiety and Depression |
| Zeraati et al | - IFN-γ and IL-17A levels - increased IL-10 - hippocampal BDNF - learning and memory impairments - anxiety-related   behaviors   - depression-related symptoms - hippocampal TNF-α and IL-1β | -Y maze  -Morris water maze  -Novelty-suppressed feeding test  -Open field  -Forced swim test  -Elevated plus maze –Light/dark box  -Cytokines and BDNF measurements |  | gavage | Using antibiotics, Gut microbiota depletion | Pregnant-C57BL6 mice/160 | Effect of gut microbiota depletion on immunological  and neurobehavioral responses in a mouse model of MS |
| Murray et al. 2019 | - LPS-induced sickness behavior - LPS-induced changes in body weight - LPS-induced increases in pro- and anti-inflammatory peripheral cytokines - central cytokine mRNA expression in hippocampus - LPS-induced changes to in the gut microbiota   altered toll-like receptor-4 activity | -Sickness monitoring  -Multiplex luminex immunoassay  -Real-time qPCR  -Amplicon sequencing  -Elevated plus maze  -Open field test  -Rotarod test  -Forced swim test  -Restraint stress  -Tissue collection  -Immunohistochemistry  -Image analysis  -Cell counting |  | Dissolve in drinking water | L. lactis  L. cremoris  L. diacetylactis  L. acidophilus | CD-1 mice/160 | Effect of probiotic on depression- and anxiety-like behaviors |
| Yang et al 2019 | - hippocampal microgliosis - proinflammatory cytokines (tumor necrosis factor-α (TNF-α), interleukin-1β (IL-1β) and IL-6) expression - neuronal   development   - synapse plasticity - learning and memory | -Contextual fear conditioning test  -Barnes circular maze test  -Measurement of hippocampal cytokines and serum endotoxin | 4 weeks | gavage | Akkermansia muciniphila | Juvenile C57BL-6J mice/- | Effect of early-life high-fat diet on hippocampal  development and cognitive functions via regulation of gut  commensal Akkermansia muciniphila |
| Choi et al. 2019 | - BDNF Expression in Cultured Hippocampal Neurons - depressant behavior | -Quantitative real-time PCR  -Western blot analysis  -Sociability test  -Tail suspension test  -Forced swim test |  | IP | L. plantarum | C57BL-6J mice/- | Effect of extracellular vesicles derived from L.  plantarum on BDNF Expression in  Cultured Hippocampal Neurons |
| 28 Watcharin et al 2019 | - depressant behavior - BDNF gene expression in hippocampus - Lactobacillus population | -Open field test-Elevated plus maze-Forced swimming test-Fecal library preparation and DNA sequencing-Hormone measurement-Quantitative real-time PCR | 2 weeks | supplementation, | L. reuteri | C57BL-6J mice/30 | Effect of ovarian progesterone on depression and anxiety-like behaviors by increasing the Lactobacillus population of gut microbiota |
| [Wang](https://www.ncbi.nlm.nih.gov/pubmed/?term=Wang H%5BAuthor%5D&cauthor=true&cauthor_uid=30998517) et al. 2019 | - beta-3 band in the hippocampus - mental fatigue | -CBG questionnaires-MEG recording | 4 weeks | Oral | B. longum 1714™ | Clinical/40 | Effect of B. longum 1714™ strain on brain  activity during social stress |
| Meng et al 2019 | microglia-mediated neuroinflammationROS production in the hippocampusexpression of antioxidant enzyme methionine sulfoxide reductase | -Fear conditioning test -Open field test  -Western blot analysis  -Hydrogen peroxide assay  -Immunofluorescent study  -Circulating TMAO and proinflammatory cytokine measurements | 3 weeks | in drinking water | trimethylamine N-oxide (TMAO) | Aged F344xBN F1 rats/56 | Effect of trimethylamine N-oxide on cognitive dysfunction |
| Jang et al. 2019 | NF-κB activationoccurrence and development of anxiety/depressioninfiltration of Iba1+ and LPS+/CD11b+ cells (activated microglia) into the hippocampushippocampal BDNF expression | -Elevated plus maze-Immunobloting-ELISA -Immunofluorescence Assay-Quantitative polymerase chain reaction (qPCR)-Limulus amoebocyte lysate assay | 5 days | gastric gavages | L. reuteri NK33B. adolescentis NK98 | C57BL-6 mice/42 | Effects of L. reuteri NK33 and B. adolescentis NK98 on immobilization stress-induced anxiety/depression and colitis |
| Pan et al 2019 | - anxiolytic-like behavior in GF mice - expression of CREB1 in SPF mice | -PCR microarrays  -Bioinformatics analysis  -Western blotting validation |  |  | Germ free | Balb-c GF mice/68 | Effect of gut microbiota absence on anxiolytic behaviors and monoamine neurotransmitters system |
| 34 Jiah et al 2019 | - Ventral hippocampus (vHPC) and plasma markers of inflammation - microglial density and IL-1β expression in the vHPC - depression-like behaviors | -Forced swim test  -Immunoassays | 6 days | oral gavage | fecal transplantation | Sprague–Dawley rats/- | Effect of gut microbiome on depressive-type behaviors and in inflammatory processes |
| [Mohammadi](https://www.ncbi.nlm.nih.gov/pubmed/?term=Mohammadi G%5BAuthor%5D&cauthor=true&cauthor_uid=30810993) et al. 2018 | - Bax and Bax/Bcl-2 ratio - Bcl-2 expression - caspase-3 activation - expression of procaspase-3 - cleaved caspase-3 - hippocampal apoptosis | -Western blotting of hippocampal tissues | 2 weeks | gastric gavages | L. helveticus R0052  B. longum R0175 | Wistar rat/40 | Effect of L. helveticus R0052 and B. longum R0175 mixture on hippocampal apoptosis |
| Bistoletti et al 2019 | - BDNF and TrkB protein levels in hippocampus | -Immunofluorescence  -Real-time quantitative RT-PCR  -Western immunoblot analysis | 2-week | gavage | Antibiotic treatment | Juvenile C57BL-6J mice/- | Effect of antibiotic treatment on BDNF and TrkB expression |
| Zheng et al 2019 | - Glutamate - higher glutamine - GABA in hippocampus - SCZ-relevant behaviors | -Open-field test  -Sociability and social novelty preference test  -Y-maze  -Forced swimming test  -Comparisons of metabolite profiles from the FMT model  -Metagenomic analysis of fecal samples | Single dose | gavaged | schizophrenia gut microbiome transplantation | GF Kunming  Mice/- | Effect of gut microbiome from patients with schizophrenia  on glutamate-glutamine-GABA cycle and  schizophrenia-relevant behaviors |
| Jang et al. 2019 | - HFD-induced NF-κB activation - HFD-suppressed AMP-activated protein kinase (AMPK) activation - SIRT-1 expression in the liver - HFD-induced anxiety-like behaviors - BDNF expression - NF-κB activation in hippocampus | -Elevated plus maze  -Marble-burying tasks, -Myeloperoxidase activity assay  -Determination of LPS in the Blood, liver and feces  -Determination of total cholesterol (TC), HDL, triglyceride (TG), aspartate Transaminase (AST), and alanine Transaminase (ALT) -Immunoblotting  -ELISA  -Immunofluorescence Assay  -Quantitative real time-PCR  -Pyrosequencing | 8 weeks | oral | L. sakei OK67  tyndallized OK67 heat-stable L. sakei PK16 | C57BL-6 mice/40 | effects of heat-labile L. sakei OK67, tyndallized OK67 (tOK67), and heat-stable L. sakei PK16 on HFD-induced obesity and anxiety |
| [Romo-Araiza](https://www.ncbi.nlm.nih.gov/pubmed/?term=Romo-Araiza A%5BAuthor%5D&cauthor=true&cauthor_uid=30618722) et al. 2018 | - performance in MWM - concentrations of pro-inflammatory cytokines (p < 0.01) - IL-1β level - BDNF level - butyrate level - N-methyl-D-aspartate receptor (NMDA)/AMPA ratio and - long-term potentiation | -MWM-Pavlovian autoshaping test-Food-magazine training-Autoshaping training-ELISA-Fecal butyrate concentrations-Electrophysiology Recordings | 5 weeks | gastric gavages | E. faecium+ inulin | Sprague-Dawley rat/52 | Effect of probiotics and prebiotics on memory |
| Abraham et al. 2019 | - number of beta-amyloid plaques in hippocampus | -Open field test  -MWM  -Spontaneous alteration tests  -Immunofluorescent labeling  -β-amyloid and OGG1 staining  -Double labeling 6E10 and Iba1 | 20 weeks |  | B. longum  L. acidophilus | APP/PS1 transgenic mice/32 | Effect of probiotic on AD development |
| Li et al. 2018 | - anxiety- and depressive-like behaviors - Lactobacillus abundance   reversed the CMS-induced immune changes in the hippocampus   - hippocampal TNF-a level - IDO1 protein levels in the hippocampus | **-Sucrose preference test****-Elevated plus maze****-Forced swim test****-Hippocampal cytokine measurements****-Western blotting****-Fecal microbiota analysis** | 4 weeks | gastric gavages | L. helveticus R0052, L. plantarum R1012 B. longum R0175 | C57BL-6 mice/48 | **Effect of probiotic on behavioral deficits Induced by chronic mild stress** |
| Jang et al. 2018 | - BDNF expression - NF-κB activation in hippocampus - infiltrations of Iba+ and CD11b+/CD45+ into hippocampus particularly CA3 region - anxiety-like behaviors and biomarkers | -Elevated plus-maze -Immunoblotting-ELISA-Immunofluorescence assay-Quantitative polymerase chain reaction (qPCR)-Pyrosequencing-Limulus amoebocyte lysate (LAL) assay | 4 days | gastric gavages | L. johnsonii  L. plantarum | C57BL-6 mice/32 | Effect of probiotics on anxiety-like behaviors and biomarkers |
| Lee et al. 2018 | - BDNF expression - CREB expression phosphorylation in hippocampus - TNF-α expression - NF-κB activation - BDNF expression | -Passive avoidance test  -Y-maze task  -Analysis of NF-κB activation and BDNF expression  -ELISA  -Acetylcholinesterase activity | 5 days | gastric gavages | L. johnsonii CJLJ103 | ICR mice  SH-SY5Y and BV-2 cells/18 | Effect of L. johnsonii CJLJ103 on Scopolamine-Induced  Memory Impairment |
| Huang et al. 2018 | - anxiety-like behaviors - memory impairment - neural monoamines levels in the striatum, hippocampus, and serum - anti-oxidative enzymes including superoxide dismutase (SOD) and glutathione peroxidase (GPx) - TNF-α - monocyte chemotactic protein-1 (MCP1) - interleukin (IL)-10 - inflammation | -Open field test  -MWM  -Quantification of neuronal amines and their metabolites  -ELISA  -Antioxidative enzyme activities | 28 weeks | gastric gavages | L. paracasei PS23 | SAMP8 mice/12 | Effect of L. paracasei PS23 on progression of age-related cognitive decline |
| Xu et al. 2018 | depressive behaviorsneuronal cell injury in hippocampal CA3 regions.MDA levelsSOD activityp-AKT and Bcl-2 levels in the hippocampusconcentrations of Baxcleaved caspase-3neural apoptosis | -Measurement of feces parameters and intestinal transit ratio-Forced swimming test-Tail suspension test-Assessment of MDA level and T-SOD activity-Protein preparation and western blotting-Tissue preparation and cresyl violet stain | 14 days | gastric gavages | bifidobacteria lactobacillus laccoccus  yeast | ICR mice/36 | Effect of probiotics on depressive behaviors induced by constipation |
| Jang et al 2018 | - anxiety and colitis - blood corticosterone - IL-6 - lipopolysaccharide levels - recruitment of microglia (Iba1+), monocytes   (CD11b+/CD45+), and dendritic cells (CD11b+/CD11c+) to the hippocampus  population of apoptotic neuron cells  (caspase-3+/NeuN+)   - NF-κB activation - IL-1β expression - TNF-α expression - gut membrane permeability | -EPM  -Light/dark transition and marble-burying tasks  -Immunoblotting  -ELISA  -qPCR  -Immunofluorescence  -Flow cytometry  -Pyrosequencing  -Limulus amoebocyte lysate assay  -In vivo intestinal permeability assay of FITC-dextran | 5 days | gavage | fecal microbiota transplantation of mice treated with ampicillin | C57BL-6 mice/16 | Evidence for interplay among antibacterial-induced gut microbiota disturbance, neuro-inflammation, and anxiety in mice |
| Christine al. 2018 | - anxiety - BDNF expression - NF-κB activation in hippocampus - blood levels of corticosterone - blood levels of IL-6 - blood levels of TNF-α - iNOS and COX-2 expression | -6-Hz psychomotor seizure assay  -Glucose measurements  -16S rDNA microbiota profiling  -Microbiota conventionalization  -Kcna1 seizure recordings  -Colonic lumenal and serum metabolomics  -Hippocampal metabolomics  -Cross-feeding in vitro assay  -GGT activity assay  -Intestinal permeability assay | 14 days | gastric gavages | L. reuteri  L. johnsonii  L. plantarum  L. rhamnosus | -SPF wild type Swiss Webster mice  -GF wildtype Swiss Webster mice  -SPF C3HeB/FeJ KCNA1 KO mice | The anti-seizure effects of Akkermansia and Parabacteroides |
| Athari Nik Azm et al. 2018 | - spatial memory - malondialdehyde levels - superoxide dismutase activity - memory deficit | -MWM  -Amyloid plaque detection by congo red staining  -Detection of superoxide dismutase and catalase activities and malondialdehyde levels in hippocampal tissue  -Detection of bacteria counts in stool samples | 8 weeks | Dissolve in drinking water | L. acidophilus)  L. fermentum  B. lactis  B. longum | Wistar rat /- | Effect of Lactobacilli and bifidobacteria on memory and learning deficits and oxidative stress |
| Zhang et al. 2018 | - spatial learning and memory - astrocytes and microglia activity in hippocampus. - mRNA and protein expression of inflammatory cytokines (TNF-α, IL-1β, IL-6, and IL-8) | -Open field test  -MWM  -Passive avoidance test  -RNA extraction and -RT-PCR analysis  -ELISA  -Western blot  -Immunohistochemistry | 7 days |  | Porphyromonas gingivalis-LPS | C57BL-6 mice/60 | Effect of Porphyromonas gingivalis lipopolysaccharide on cognitive dysfunction |
| [Neufeld](https://www.ncbi.nlm.nih.gov/pubmed/?term=McVey Neufeld KA%5BAuthor%5D&cauthor=true&cauthor_uid=29173065) et al. 2019 | - stress - anxiety - hippocampal-dependent learning | -Open field  -MWM  -Quantitative real-time PCR |  | In their diet | L. rhamnosus GG |  | Neurobehavioural effects of L. rhamnosus GG alone and in combination with prebiotics polydextrose and galacto -oligosaccharide in male rats exposed to early-life stress |
| Agusti et al. 2018 | - obesity-induced upregulation of TLR2 protein or gene expression in the intestine (p < 0.010) and the hippocampus (p < 0.050) and restored the alterations of 5-HT levels in the hippocampus | -Hormonal and metabolic parameter analyses  -RT-qPCR analysis  -Determination of corticosterone levels  -Sucrose and saccharin preference test  -Light–dark box test  -Open field test  -Neurotransmitter analysis  -Forced swimming test  -Immunostaining | 13 weeks | gastric gavages | B. pseudocatenulatum CECT 7765 | C57BL-6 mice/40 | Effect of B. pseudocatenulatum CECT 7765 on neuroendocrine alterations |
| [Jeong](https://www.ncbi.nlm.nih.gov/pubmed/?term=Jeong JJ%5BAuthor%5D&cauthor=true&cauthor_uid=27824273) et al. 2016 | - expression of inflammatory markers, such as myeloperoxidase, tumour necrosis factor (TNF), and interleukin (IL)-1β - NF-κB activation - expression of senescence markers p16, p53, and SAMHD1 in the colon and the hippocampus - expression of brain-derived neurotrophic factor | -Y-maze task  -Assay of myeloperoxidase activity  -ELISA  -immunoblotting | 8 weeks | oral | L. brevis OW38 | C57BL-6J mice/24 | Anti-inflammaging effects of Lactobacillus brevis OW38 in aged mice |
| Sun et al. 2016 | - histopathologic changes in the CA1 region of hippocampus. - cognitive impairment - cell damage - apoptosis - p-Akt expression - caspase-3 expression - neuronal apoptosis - fecal microbiota diversity and changes of fecal microbiota composition | -MWM  -Histology  -Western blot  -PCR-DGGE analysis  -qPCR | 6 weeks | gastric gavages | C. butyricum | C57BL-6 mice/36 | Effect of C. butyricum on cerebral ischemia/reperfusion injury in diabetic mice |
| Liu et al. 2015 | - cognitive dysfunction   histopathological changes   - BDNF level - Bcl-2 level - Bax level - Neuronal apoptosis in hippocampus Moreover, C. - gut microbiota regulation | -MWM  -PCR-DGGE analysis  -Ultrastructure analysis  -Histology analysis  -Western blot  -Immunohistochemistry  -Butyrate assay | 6 weeks | gastric gavages | C. butyricum | ICR mice/60 | Neuroprotective effects of C. butyricum against  vascular dementia |
| Liu et al. 2016 | - total distance traveled in the open field test - the time spent in the closed arm in the elevated plus maze test - levels of both serotonin and dopamine in the striatum, but not in the prefrontal cortex or hippocampus | -Blood biochemistry  -Histopathological examination  -Open field test  -Elevated plus maze  -Forced swim test  -Serum corticosterone levels  -Quantification of brain monoamines and their metabolites -Liquid chromatography | 16 days | oral | L. plantarum PS128 | GF C57BL-6JNarl mice/30 | Effect of L. plantarum on alteration of behavior and monoamine levels |
| Liang et al. 2015 | - anxiety and depression and cognitive dysfunction - plasma corticosterone - adrenocorticotropic hormone (ACTH) levels - plasma interleukin-10 (IL-10) levels - hippocampal   serotonin (5-HT) and norepinephrine (NE) levels   - hippocampal brain-derived neurotrophic factor (BDNF) mRNA expression | -Sucrose  preference test  -Elevated-plus maze test  -Open-field test  -Object recognition test  -Object placement  test  -Biochemical analysis | 3 weeks | Dissolve in drinking water | L. helveticus NS8 | specific-pathogen-free (SPF) Sprague–  Dawley rats/32 | Effect of L. helveticus NS8 on behavioral cognition and biochemical aberrations |
| Wang et al 2015 | - normalization of gut microbiota composition - ampicillin-induced inflammation - mineralocorticoid and N-methyl-D-aspartate receptors in hippocampus - anxiety-like behavior - ampicillin-induced impairment in memory retention | -Elevated plus maze  -MWM  -Corticosterone analysis | 30 days | Dissolve in drinking water | L. fermentum NS9 | Sprague-Dawley rats/30 | Effect of L. fermentum NS9 on antibiotic induced physiological and psychological abnormalities |
| Distrutti et al. 2014 | - abundance of Actinobacteria and Bacterioidetes - expression of a large group of genes that impact on inflammatory and neuronal plasticity processes - age-related deficit in LTP - markers of microglial activation - expression of BDNF and synapsin. | -Analysis of intestinal microbiota  -Microarray analysis  -Microarray validation by PCR  -LTP  -RT-PCR  -Western blot | 6 weeks | maple syrup | VSL#3 | Wistar rats/- | Effect of VSL#3 on modulation of intestinal microbiota and rain gene expression |
| Christina et al. 2013 | - anxiety-like behavior - brain levels of corticosterone | -Barnes maze  -Histology  -Immunoassays  -Terminal restriction fragment length polymorphism (T-RFLP)  -Short chain fatty acid (SCFA) analysis | 3 weeks | gastric gavages | L. helveticus | Mice/- | Effects of L. helveticus on murine behavior with alterations in the gut microbiome |
| Wei et al. 2019 | - Abnormal behavioral changes - brain-derived neurotropic factor, mineralocorticoid, and glucocorticoid receptors in hippocampus   In addition, also reverses  corticosterone-reduced   - serotonin levels in hippocampus using live PS23 - dopamine levels in hippocampus using heat-killed PS23 | -Open field test  -Forced swim test  -Sucrose preference test  -Serum corticosterone measurement  -Brain protein analysis  -Brain monoamine analysis | 41 days | gastric gavages | L. paracasei PS23 | C57BL-6J mice/40 | Antidepressant effect of live and heat-killed L. paracasei PS23 |
| Shang-Tse Ho et al 2019 | - memory and learning abilities - apoptosis pattern in the hippocampus of aging mice - activities of the antioxidant enzymes superoxide dismutase and catalase - production of malondialdehyde - antioxidant-related proteins nuclear factor erythroid 2-related factor 2 (Nrf2) and heme oxygenase-1 (HO-1) | -MWM  -Reference memory task  -Working memory task  -Detection of antioxidant  -Biochemical parameters  =Immunohistochemistry staining  -Western blot analysis  -Quantitation of short-chain fatty acids  -Contents in feces sample | 12 weeks | oral | L. paracasei  L. plantarum  S. thermophilus | C57B-CL6 mice/15 | Improving effect of a probiotic mixture on memory and learning  abilities in d-galactose–treated aging mice |
| Corpuz et al. 2018 | - cognitive performance in the Barnes maze and passive avoidance tests. - serotonin level - protein expression of BDNF - cAMP response element binding protein (CREB) - phosphorylated   CREB | -Barnes maze test  -Passive avoidance test  -Y-maze test  -ELISA  -Q-PCR  -Western blotting  -Immunostaining | 29 weeks | In their diet | L. paracasei K71 | SAMP8 mice/36 | Effect of  L. paracasei K71 supplementation on age-related cognitive decline |
| Ait-Belgnaoui et al. 2018 | - chronic stress - expression of the glucocorticoid receptor mRNA which correlated with the normalization of stress response | -Plasma stress hormone analysis  -Gene expression studies  -Electromyography recording | 2 weeks | gastric gavages | B. longum  L. helveticus | C57BL-6J mice/64 | Effect of B. longum and L. helveticus on stress-related visceral hypersensitivity |
| Neufeld et al. 2017 | - anxiety-like behaviour - hippocampal-dependent learning | -Open field  -MWM  -Q-real-time PCR | 7 weeks | In their diet | L. rhamnosus GG | Sprague-Dawley  Rats/30 | Neuro-behavioral effect of L. rhamnosus GG on polydextrose and  Galacto-oligosaccharide |
| O'Hagan et al. 2017 | - spatial navigation and robust - long-term object recognition memory and short-term memory - neural signalling modulation | -MWM  -Object recognition  -1H NMR spectroscopic analysis |  | diet | L. acidophilus  L. acidophilus  B. bifidum  B. lactis | Lister Hooded rats/32 | Effect of Long-term multi-species Lactobacillus and Bifidobacterium dietary supplement  on memory and changes regional brain metabolites |
| Allen et al. 2016 | - stress - hippocampus-dependent visuospatial memory performance - memory | -Questionnaires  cambridge neuropsychological test  -Automated battery as cognitive tasks  -Neurocognitive assessment  -Salivary cortisol analysis | 4 weeks | Oral | B. longum 1714 | Clinical trial/22 | Effect of B. longum 1714 as a translational psychobiotic:  modulation of stress, electrophysiology and neurocognition |
| Sun et al. 2016 | - neurological deficit improvement - histopathological change in the hippocampus - MDA contents - SOD activities - expression of Caspase-3 and Bax - Bcl-2/Bax ratio - butyrate contents in the brain | -Neurological deficit score  -Histopathology  -SOD activity analysis  -MDA contents determination  -Western blot  -Immunohistochemistry | 2 weeks | gastric gavages | C. butyricum | ICR mice/- | Effect of C. butyricum pretreatment on cerebral ischemia/reperfusion injury |
|  |  |  |  |  |  |  |
| Jeong et al. 2015 | - blood triglyceride - HDL cholesterol - double cortin and BDNF expression - expression of p16, p53, and cyclooxygenase-2, the phosphorylation of Akt and mTOR - activation of NF-κB in hippocampus | -Y-maze  -MWM  -ELISA  -Blood triglyceride, total cholesterol and HDL cholesterol analyzing | 8 weeks | Oral | L. curvatus  L. plantarum . | Fischer 344 rats/24 | Effect of probiotic mixture KF on age-dependent memory deficit and  lipidemia |
| Davari et al. 2013 | - spatial memory - declined basic synaptic transmission - hippocampal long-term potentiation (LTP) - activation of superoxide dismutase - insulin level of serum - both the glucose level of serum and the 8-OHdG factor | -MWM  -Electrophysiological recording  -Biochemical measurements | 8 weeks | dissolved in drinking water | L. acidophilus  B. lactis  L. fermentum | Wistar rats/40 | Effect of probiotics treatment on diabetes-induced impairment of synaptic activity and cognitive function |
| O’Sullivan et al. 2011 | - BDNF total variants in controls | -PCR analysis  -In situ hybridization |  | gavage. | B. breve 6330 | Sprague Dawley rats/40 | Effect of B. breve 6330 on BDNF Expression in the Hippocampus |
| Sousa et al. 2008 | - body weight - cytoplasmic stain in neurons of the cerebral cortex, thalamus, hypothalamus, hippocampus and, to lesser degree, in the cells of the choroid   plexus   - leptin and leptin receptors |  |  | Icv???? | L. acidophilus | Sprague Dawley rats/38 | Effect of L. acidophilus supernatants on body weight and  leptin expression |
| Tian et al. 2019 | - depressive behaviors - level of 5-hydroxytryptamine - BDNF concentration - serum corticosterone level - depression - chronic stress - microbial dysbiosis | -Forced swim test  -Tail suspension test  -SAucrose preference test  -Open field test  -Elevated plus maze  -Light/dark box test  -Step down test | 5 weeks | oral | B. longum ..  B. infantis E41  B. breve M2CF22M7 |  | Effect of Bifidobacterium on  depression and related microbiota dysbiosis |
| Chunchai et al 2018 | - inflammation - hippocampal plasticity and - brain itochondrial dysfunction - hippocampal oxidative stress and apoptosis - microglial activation - cognitive function | -Metabolic parameters determination  -Q-real-time PCR analysis  -Extracellular recordings  -Brain mitochondrial function  -Immunoblotting  -Immunofluorescent  -Microglial morphology  -MWM  -Gut microbiota analysis | 12 weeks | Oral feeding | L. paracasei HII01 | Wistar rats/- | Effect of prebiotics, probiotics, or synbiotics on cognitive restoration |
| Matthew et al. 2018 | - anti-inflammatory immunophenotype in hippocampus - interleukin 4, Cd200r1, and Mrc1 mRNA expression - IL4 protein - Basal levels of genes (Nlrp3 and Nfkbia) involved in microglial priming - stress-induced hippocampal microglial priming and HMGB1, - anxiety-like behavior | -Inescapable tail shock  -Serum corticosterone assay  -Ex vivo immune stimulation of hippocampal microglia with LPS  -Real time RT-PCR  -ELISA  -Juvenile social exploration | -21 d, -14 d  and -7 d | subcutaneous (s.c.) immunizations | Mycobacterium vaccae | Sprague-Dawley rats/- | Effect of M. vaccae on attenuation of stress-induced microglial priming, alarmins and anxiety like behavior |
| Ramalho et al. 2019 | - depressive- and anxiety-like behavior - immobility time in the tail suspension test and forced swim test - percent of time spent in the open arms on the elevated plus maze | -Antimicrobial activity  -Antibiotic susceptibility test  -Determination of in vitro antioxidant activities  -Open field  -Elevated plus maze -Tail suspension test  Forced swim test  -Reactive species quantification  -Ferric reducing antioxidant power assay  -Quantification of fecal LAB | 4 weeks | oral | L. lactis subsp. cremoris LL95 | C57BL-6 mice and invitro/12 | Effect of L. lactis subsp. cremoris LL95 on antioxidant potential and behavior |
| Lee et al 2018 | - memory impairment - hippocampal BDNF expression - hippocampal NF-κB activation - blood LPS levels - gut microbiota composition disturbance | -Y-maze  Noble object recognition test  -Passive avoidance tasks  -Myeloperoxidase activity  -Immunoblotting  -Fecal microbiota composition analysis | 3 , 5 days | oral | L. plantarum C29 | C57BL-6 mice/- | Effect of L. plantarum C29 on TNBS-Induced Memory  Impairment |
| Lin et al. 2019 | - survival and growth - enteritis - abundance of intestinal Lactobacillus | -16S rDNA sequencing analysis  -Microbial analysis  -Immunocytokine analysis  -Transmission electron microscopy | 45 days | Drinking water | L. acidophilus  L. plantarum  L. rhamnosus | Clinical/- | Effect of Lactobacillus spp. as probiotics on prevention and treatment of enteritis in  the lined seahorse |
| Laureano-Melo et al. 2019 | - behavioral parameters improvement - BDNF expression - hippocampal GABAergic system modulation | -RNA analysis  -Open field test  -Light-dark box test  -Elevated plus maze test  -Tail suspension test |  | gavage | L. paracasei DTA 83 | Swiss Webster mice/12 | Effect of maternal supplementation with L. paracasei DTA 83 on  emotional behavior |
| Abildgaard et al. 2017 | - depressive-like behaviour - IFNγ, IL2 and IL4 at the expense of TNFα and IL6. - hippocampal transcript levels of factors involved in HPA axis regulation - level of indole-3-propionic acid, apotential neuroprotective agent | -Barnes maze  -Forced swim test  -Open field test  -Oral glucose tolerance test  -Anti-CD3/28 stimulation of PBMC  -Measurement of cytokines  -Plasma endotoxin  -Real-time qPCR  -Metabolomics | 5 weeks | In their diet | B. bifidum W23  B. lactis W52  L. acidophilus W37 L. brevis W63  L. casei W56  L. salivarius W24  L. Lactis W19  L. Lactis W58 | Sprague-Dawley rats/40 | Effect of probiotic on depressive-like behaviour |
| Rezaei Asl et al 2019 | - maze navigation improvement - LTP induction - balance of the anti-oxidant/oxidant biomarkers | -MWM  -Electrophysiological experiments  -Fecal bacteria quantification  -Measurement of the biomarkers  -Histological examinations | 8 weeks | gavage | L. acidophilus  B. bifidum  B. longum | Wistar rats/- | Effect of probiotic on spatial cognitive performance  and synaptic plasticity |
| Divyashri et al. 2015 | - ROS in hippocampus - oxygen radical absorbance - anti-inflammatory effect - TNF-a - IL-10 levels - endogenous   oxidative markers | -Open field test  -Elevated plus  maze  -Biochemical analysis  -Generation of reactive oxygen species  -Determination of reduced glutathione  -Determination of protein carbonyls  - Enzyme activity  -Quantification of c-aminobutyric acid (GABA) and dopamine  (DA) levels | 4 weeks | Gavage | E. faecium CFR 3003 | CFT-Swiss mice/24 | Antioxidant, anti-inflammatory  and neuromodulatory effects of E.  faecium CFR 3003 |
| Hadizadeh et al 2018 | - behavioral parameters improvement - serum corticosterone concentration | -Elevated plus-maze  -MWM  -Serum level of corticosterone | 2 weeks | dissolved in drinking water | L. acidophilus  B. lactis  L. fermentum | Wistar rats/36 | Effect of probiotic supplementation on cognitive function and  the anxiety-like behaviors |
| [Rahmati](https://link.springer.com/article/10.1007%2Fs11033-019-04949-7#auth-1) et al 2019 | - apoptotic cells - neuronal death in the CA1, CA3 and DG of the hippocampus - reduced spatial memory impairment - neurological dysfunction - hippocampus damage - spatial learning and memory deficit - apoptosis | -TUNEL assay-Cresyl violet staining-Radial arm water maze-Measurement of the hippocampus MDA, FRAP content and BDNF level | 3 weeks | orally | L. caseiL. acidophilusL. rhamnosusL. bulgaricusB. breveB. longumS. thermophilus | Swiss albino mice/50 | Effect of probiotic supplementation on hippocampus injury and spatial learning and memory impairments |
| Zhu 2019 | - behavioral abnormalities - learning and memory impairment - asal extracellular dopamine - tryptophan degradation |  | 3 weeks |  | SCZ microbiota transplantation | C57BL-6J mice/- | Effect of microbiota transplantation from drug-free patients with schizophrenia on schizophrenia-like abnormal behaviors and dysregulated kynurenine metabolism |
| xiao et al 2020 | - memory dysfunction - neural re-damages - inflammation - IL-6 expression | -MWM -Y-maze -Spine counting-Microbiome analysis-Immunoblots and immunostaining-ELISA | 5 weeks | Drinking water | B.longumL. acidophilusL. fermentumL. helveticusL. paracaseiL. rhamnosusS.thermophilus | Sprague-Dawley (SD) rats/- | Effect of Long-term probiotic intervention on memory dysfunction |
| [Tahmasebi](https://www.ncbi.nlm.nih.gov/pubmed/?term=Tahmasebi S%5BAuthor%5D&cauthor=true&cauthor_uid=32028126) et al 2020 | - kindling development - escape latency and traveled path In MWM test - Population spike (PS)-LTP | -Kindling  -Field potential recordings | 6 weeks | Gavage | L. casei  L. acidophilus  B. bifidum | Wiastar rat/44 | Effect of probiotics and Nigella sativa extract on behavioral and electrophysiological effects of PTZ-induced chemical kindling |
| [Wang](https://www.ncbi.nlm.nih.gov/pubmed/?term=Wang QJ%5BAuthor%5D&cauthor=true&cauthor_uid=31907339) et al 2020 | - Cognition - Αβ levels in the hippocampus - neuronal integrity and plasticity - TMAO synthesis - Neuro-inflammation - intestinal microbiota remodeling | -Spontaneous locomotor activity test  -Nest building test  -Novel object recognition test  -MWM  -Shuttle-box test  -Electrophysiology  -Immunofluorescence  -Nissl staining  -Golgi staining  -Soluble Aβ analysis  -ELIZA  -Quantitation of TMAO and TMA levels  -Determination of the enzymatic activity of hepatic FMO  -Multiplex bead analysis  -Metagenomic analyses | 12 weeks | Gavage | L. plantarum ATCC 8014 | C57BL-6J mice/105 | Effect of concomitant memantine and L. plantarum  on cognitive impairments |
| Talani et al. 2020 | - hippocampal - dendritic spines’ density of hippocampal CA1 pyramidal neurons - LTP formation on the CA1 hippocampal region - cognitive performance - locomotion activity impairment | -Immunoblot analysis-Golgi-Cox staining and dendritic spines morphology-Image analysis-Spine quantification and neuronal morphology-Electrophysiology-Open field-Barnes maze-Novel object recognition | 4 weeks | gavage | B. longum  B. breve.  B. infantis | Sprague Dawley rats/- | Effect of Bifidobacteria on hippocampal plasticity and cognitive behavior |
| Tsukahara et al 2019 | - mRNA levels of bdnf, nt-3, and GABAR - habituation ability and behavior improvement | -Open field  -Social interaction  -Sucrose preference  -Tail suspension  -Forced swimming test  -mRNA level analysis in the hippocampus | 3 weeks | oral | L. plantarum strain SNK12 | C57BL-6JJmsSlc (B6) and Slc:ICR mice/74 | effect of oral supplementation of L. plantarum strain SNK12 on mRNA levels of neurotrophic factors and GABA receptors |
| [Mohammed](https://www.sciencedirect.com/science/article/pii/S0031938419310972?via%3Dihub#!) et al. 2020 | - cognitive deficits - hippocampal TLR4 expression - BDNF expression and protein - number of viable cells - pyramidal cells in hippocampus | -Novel object recognition task  -MWM  -Real-time PCR  -ELISA  -Histological studies | 12 weeks | Gavage | L. Plantarum | Wister rats/48 | Effect of probiotics on modulation of hippocampal TLR4/BDNF signal pathway |
| [Li](https://www.ncbi.nlm.nih.gov/pubmed/?term=Li Y%5BAuthor%5D&cauthor=true&cauthor_uid=32357144) et al 2020 | - cognitive behavior impairment - regional homogeneity in medial prefrontal cortex and hippocampus - dendritic spines - expression of BDNF - N-methyl-D-aspartate receptor NR1 subunit - Synaptophysin - expression of advanced glycation end products (AGEs) and receptor for AGEs (RAGE). - levels of pro-inflammatory cytokines - oxidative stress | -Delayed matching to position (DMTP) task  -16S rRNA gene sequencing  -Resting-state functional magnetic resonance imaging  -Golgi staining  -Transmission electron microscopy  -Western blot  -Immunohistochemistry  -ELISA  -Analysis of oxidative stress level | once a day for three days, then twice a week for two months | oral gavage | Age-related microbiota transplantation | SD rat/- | Effect of ge-related shifts in gut microbiota on cognitive decline |
| [Miranda](https://pubmed.ncbi.nlm.nih.gov/?term=de+Miranda+AS&cauthor_id=32580070) et al 2020 | - IL- 2 - IL-4 - IL-6 - IL-10 - IL-17A - TNF-α | -Open field  -ELISA  -Cytometric bead array (CBA) |  | intraperitoneal injection  of GBR12909 | germ-free (GF) | germ-free (GF) Swiss mice/- | Role of gut microbiota in the GBR12909 model of mania-like behavior in mice |
| Shen et al 2020 | - escape latency - time to find platform - number of platform crossings - memory ability - athletic ability - activation of microglia - nerve cell damage - expression of inflammatory factors in hippocampus | -MWM  -Immunofluorescence (IF) staining  -Immunohistochemistry  -Nissl staining in mouse hippocampus  -Western blot | 4 weeks |  | fecal microbiota transplantation | APP/PS1 double transgenic C57BL-6 mice/10 | effect of gut microbiota on neuroinflammation in Alzheimer's disease |
| [D'Amato](https://pubmed.ncbi.nlm.nih.gov/?term=D'Amato+A&cauthor_id=33004079) et al 2020 | - spatial learning and memory - anxiety - synaptic plasticity and neurotransmission in hippocampus - microglia cells of hippocampus | -Amplicon sequencing-16S rRNA analysis-Metabolomics analysis -Barnes maze test  -Open field test  -Elevated plus-maze _Liquid chromatography-MS analysis-Intestinal permeability and plasma cytokines |  | oral gavage | Faecal microbiota transplantation | C57BL-6 mice/36 | Effect of faecal microbiota transplantation from aged donor mice on spatial learning and memory |
| [Wang](https://pubmed.ncbi.nlm.nih.gov/?term=Wang+QJ&cauthor_id=31907339) et al 2020 | - cognitive impairment - Αβ levels in the hippocampus - neuronal integrity and plasticity - TMAO synthesis - Neuro-inflammation - remodeling the intestinal microbiota | -Spontaneous locomotor activity test-Novel object recognition test-MWM-Shuttle-box test-Electrophysiology-Immunofluorescence-Nissl staining-Golgi staining-Soluble Aβ analysis-Enzyme-linked immunosorbent assay-Quantitation of TMAO and TMA levels-Determination of the enzymatic activity of hepatic FMO-Multiplex bead analysis-Metagenomic analyses | 12 weeks | gavage | L. plantarum ATCC 8014 | C57BL-6J mice/60 | Effect of concomitant memantine and L. plantarum treatment on cognitive impairments |
| [Liu](https://pubmed.ncbi.nlm.nih.gov/?term=Liu+J&cauthor_id=32458851) et al 2020 | - cognition - spatial learning/memor - object recognition/memory - intestinal microbial diversity - abundance of Bacteroides and Faecalibacterium spp. Mucispirillum and Ruminiclostridium - deposition of Aβ in the cerebral cortex and hippocampus | -MWM-Novel object recognition test-16S rRNA analysis-High-throughput sequencing-ELISA-Immunohistochemistry | 20 weeks | orally by gastric intubation | Tibetan Fermented Milk and L. helveticus | APP/PS1 transgenic mice/36 | Effect of tibetan fermented milk on cognitive dysfunction by modified gut microbiota |
| [Ávila](https://pubmed.ncbi.nlm.nih.gov/?term=Marcondes+Ávila+PR&cauthor_id=32866799) et al 2020 | - IL-6 level - TNF-α level - carbonyl protein | -Open-field-Splash test-Cytokines levels-Oxidative damage parameters-Histopathological analysis | 40 days | gavage | Fecal microbiota transplantation of CMS | Wistar rat/130 | Effects of microbiota transplantation and the role of the vagus nerve in gut-brain axis in animals subjected to chronic mild stress |
| [Zhang](https://www.ncbi.nlm.nih.gov/pubmed/?term=ZHANG Y%5BAuthor%5D&cauthor=true&cauthor_uid=32775126) et al 2020 | - IL-6 - Corticosterone - abnormal expression of BDNF - microbiota dysbiosis | -ELISA  -Histopathology  -RT-PCR  -Next-generation sequencing |  | gavage | Heat-inactivated L. paracasei N1115 | pregnant Kunming mice/12 | Effect of heat-inactivated Lactobacillus paracasei N1115 on microbiota and gut-brain axis related molecules |
| [Yun](https://www.ncbi.nlm.nih.gov/pubmed/?term=Yun SW%5BAuthor%5D&cauthor=true&cauthor_uid=33182607) et al 2020 | - cognition - depressive behaviors - expression of IL-1β - populations of NF-κB^+^/Iba1^+^ and IL-1R^+^ cells - K1-suppressed population of BDNF^+^/NeuN^+^ cells in the hippocampus - colitis and gut dysbiosis | -Y-maze  -Forced swimming test -Myeloperoxidase activity assay-Immunoblotting-ELISA-Immunohistochemistry-Microbiota sequencing |  | Gavage | **L. gasseri** | Germ-free C57BL-6J mice/12 | Effect of **L. gasseri** on **E. coli**-induced cognitive impairment and depression |
| [Talani](https://pubmed.ncbi.nlm.nih.gov/?term=Talani+G&cauthor_id=31857091) et al 2020 | - hippocampal BDNF - dendritic spines - density of hippocampal CA1 pyramidal neurons - LTP formation on the CA1 hippocampal region - cognitive performance | -Immunoblot analysis  -Golgi-Cox staining and dendritic spines morphology  -Spine quantification and neuronal morphology  -Electrophysiology experiments  -Open field test  -Barnes maze  -Novel object recognition | 4 weeks. | gavage | B. longumB. breveB. infantis | Adult Sprague Dawley rat/- | Effect of bifidobacteria on hippocampal plasticity and cognitive behavior |
| [Xiao](https://www.ncbi.nlm.nih.gov/pubmed/?term=Xiao J%5BAuthor%5D&cauthor=true&cauthor_uid=32066679) et al 2020 | - memory dysfunction - neural damages - IL-6 expression - memory injuries | -MWM  -Y-maze  -Spine counting  -Microbiome analysis  -Immunoblots and immunostaining  -ELISA | 5 weeks | gavage | B. longumL. acidophilusL. fermentumL. helveticusL. paracaseiL. rhamnosusS. thermophilus | Sprague-Dawley (SD) rats/- | Effect of long-term probiotic intervention on memory dysfunction |
| [Wang](https://www.ncbi.nlm.nih.gov/pubmed/?term=Wang H%5BAuthor%5D&cauthor=true&cauthor_uid=32903531) et al 2020 | - gut development - activities of digestive enzymes - intestinal goblet cell number - DNA gene copies of Lactobacillus spp. and L. johnsonii - DNA gene copies of Enterobacteriaceae - preventive effects on intestinal permeability - inflammatory factors - tumor necrosis factor-alpha, interferon-gamma, and interleukin-10. - behavioral performance - memory-related functional proteins - synaptic plasticity - brain-derived neurotrophic factor - antioxidant capacity - apoptosis in the hippocampus | -Novel object test-T-Maze-Biochemical analysis-Real-time quantitative PCR | 4 weeks | gavage | **L. johnsonii** BS15 | ICR mice/40 | **Effect of L. johnsonii** BS15 on psychological stress–induced memory dysfunction |
| Shan et al 2020 | - behavioral abnormalities - psychomotor malaise - learning and memory impairment - tumor necrosis factorα(TNF-α) | -Open-field  -Tail suspension  -Forced swimming  -Elevated plus maze  -Tube test  -PCR  -16S rRNA analysis  -Untargeted metabolomics  -Protein analysis  -Q-real-time PCR analysis  -Immunofluorescence | 30 days | gavage | FMT of chronic stress rats | Sprague-Dawley rats/- | Effect of gut microbiome-derived lactate on anxiety-like behaviors |
| Sarkar et al 2020 | - transfer latency in EPM - instep through latency in Passive avoidance test - preference for a novel object - time spent in the specific quadrant during the probe trial in MWM test - cognition - neuronal protection in CA1 and CA3 | -Q-PCR  -Elevated plus maze  -Passive avoidance test  -MWM  -Novel object recognition -Estimation of acetylcholinesterase activity  -Estimation of myeloperoxidase activity  -Estimation of reduced glutathione  -Estimation of malondialdehyde  -Estimation of superoxide dismutase (SOD) activity  -Histopathology of brain and intestine | 14 days | gavage | S. faecalisBacillus mesenter-icus | Swiss albino mice/- | Effect of probiotics on gut dysbiosis associated decline in learning and memory |
| Liu et al 2020 | - CREB transcript | Microarray analysis  Nearby gene co-expression analysis  Functional pathway & network analysis |  |  | specific pathogen-free, germ-free and colonized germ-free mice | male SPF and GF BALB/c mice / - | Microbial regulation of alincRNA–miRNA–mRNA network in themouse hippocampus |
| [Kim](https://pubmed.ncbi.nlm.nih.gov/?term=Kim+JK&cauthor_id=32158447) et al 2020 | - cognition - anxiety - depression - BDNF expression - NF-κB activation in hippocampus - TNF-α and LPS TNF-α expression - myeloperoxidase activity - gut Proteobacteria population - fecal LPS levels | -EPM  -Tail suspension  -Forced swimming  -Y-maze  -Novel object recognition  -Barnes maze -Assay of myeloperoxidase activity-ELISA-Immunoblotting-Immunohistochemistry-Microbiota sequencing-Q-Real Time PCR-LPS Assay |  | gavage | L. mucosae NK41 | Specific pathogen-free C57BL-6J mice/- | Interplay between E. coli and L. mucosae in the occurrence of neuropsychiatric disorders |
| [Luck](https://www.ncbi.nlm.nih.gov/pubmed/?term=Luck B%5BAuthor%5D&cauthor=true&cauthor_uid=32385412) et al 2020 | - expression of synapse-promoting genes - microglia activation - synaptic density | -Immunohistochemistry-Analysis of immune-stained tissue-rRNA sequencing and analysis-Fluorescence in situ hybridization-Synaptic Plasticity qPCR array-Assessment of microglial expression of CD11b and CD45 by flow cytometry-In vivo Purkinje cell electrophysiology |  | gavage | B. dentiumB. longumB. breveB. bifidum | Germ-free (GF) Swiss Webster mice/- | Bifidobacteria shape host neural circuits during postnatal development by promoting synapse formation and microglial function |
| [Yang](https://www.ncbi.nlm.nih.gov/pubmed/?term=Yang X%5BAuthor%5D&cauthor=true&cauthor_uid=32477045) et al 2020 | - microglial numbers and activation - TNF-α - IL-1β - IL-6 - Synaptic function - BDNF | -Gut microbiota analysis  -LPS determination  -Histological staining -Immunohistochemistry  -Y-maze  -Transmission electron microscopy  -Western blot  -Q-real-time PCR | 7 days (acute) or 15 weeks (chronic) | diet | Curdlan, a bacterial polysaccharide | Sixty C57BL-6J mice/60 | Effect of curdlan on cognitive deficits induced by a high-Fat diet |
| [Xin](https://www.ncbi.nlm.nih.gov/pubmed/?term=Xin J%5BAuthor%5D&cauthor=true&cauthor_uid=33083147) et al 2020 | - gut inflammation - TNF-α - IFN-γ - anti-inflammatory cytokines (IL-10) | -Novel object recognition test-T-maze test-Biochemical evaluation -Q-real-time PCR -Immunohistochemistry | 28 days |  | L. johnsonii BS15 | ICR mice/48 | Effect of L. johnsonii BS15 on fluoride-induced memory impairment |
| [Daugé](https://www.ncbi.nlm.nih.gov/pubmed/?term=Daug%26%23x000e9%3B V%5BAuthor%5D&cauthor=true&cauthor_uid=33312120) et al 2020 | - anxiety-like behaviors - dopamine and its major metabolites - serotonin metabolites | -Novel object test  -Light-dark box test  -Elevated plus maze  -Open field test  -Forced swimming test  -Feces microbiota analysis  -LC-MS |  | Gavage | L. helveticus  B. longum  L. lactis  S. thermophilus | Fischer 344 rats/23 | Effect of probiotic mixture on anxiolytic- and anti-depressive-Like behavior |
| [Go](https://pubmed.ncbi.nlm.nih.gov/?term=Go+J&cauthor_id=33551257) et al 2021 | - immune-reactive factors - percentage of amyloid plaque deposition - microglia activation | -Novel object recognition test  -Y-maze  -Q-real-time PCR  -Immunohistochemistry  -Western blot | 10 weeks | gavage | Agathobaculum butyriciproducens | -Tg APP swe PS1dE9 transgenic mice  tg C57BL-6J ×C3H F1 hybrid mice    non-tg C57BL-6J×C3H F1 mice | Effect of A. butyriciproducens on cognitive impairment |
| Han et al 2020 | - depression-like behaviors inducement - IL-6 expression - TNF-α expression - NF-κB activation - Corticosterone - BDNF expression | -Elevated plus maze  -Forced swimming test  -Tail suspension test  -ELISA  -Immunohistochemistry  -Microbiota pyrosequencing | 5 days | gavage | L. reuteri NK33  B. adolescentis NK98 | C57BL-6 mice/35 | Effectr of L. reuteri NK33 and B. adolescentis NK98 on E. coli-induced depression and gut dysbiosis |
| [Ishii](https://pubmed.ncbi.nlm.nih.gov/?term=Ishii+T&cauthor_id=33567772) et al 2021 | - CA1 apical spine density | -Contextual fear conditioning test-cAMP assay -Q-real-time PCR -Golgi–Cox Staining-Western blot | 4 days | Gavage | B. breve | C57BL-6 mice/146 | Effect of B. breve on facilitation of hippocampal memory |
| [Zolfaghari](https://pubmed.ncbi.nlm.nih.gov/?term=Zolfaghari+SI&cauthor_id=33127463) et al 2021 | - TNF-α gene expression in hippocampus | -Passive avoidance learning test  -cDNA synthesis  -Real-Time PCR | 3 weeks | gavage | L. rhamnosusL. reuteriL. Plantarum | Adult wistar rats/56 | Effects L. rhamnosus, L. reuteri and L. Plantarum on LPS-induced memory impairment and changes in CaMKII-α and TNF-α genes expression |
| [Lee](https://www.ncbi.nlm.nih.gov/pubmed/?term=Lee KE%5BAuthor%5D&cauthor=true&cauthor_uid=33324357) et al 2020 | - cognitive function in the Y-maze, novel object recognition, and Banes maze task - NF-κB activation - Microglia activation - Apoptosis - BDNF | -Y-maze  -Novel object recognition.  -Barnes maze  -Immunofluorescence Assay  -ELISA  -Illumina iSeq sequencing |  | gavage | Ampicillin vancomycin | C57BL-6 mice/- | Effect of orally administered antibiotics vancomycin and ampicillin on cognitive impairment |
| [Han](https://www.ncbi.nlm.nih.gov/pubmed/?term=Han SK%5BAuthor%5D&cauthor=true&cauthor_uid=32224881) et al 2020 | - NF-κB activation in hippocampus - BDNF expression - Bacteroidetes population - Proteobacteria population | -Elevated plus maze  -Light/dark transition test  -Tail suspension test  -Forced swimming test  -ELISA  -Immunoblotting  -Immunofluorescence assay  -Myeloperoxidase activity assay  -Fecal microbita composition analysis | 5 days | Gavage | Bifidobacteria-Fermented Red Ginseng and Its Constituents Ginsenoside Rd and Protopanaxatriol | C57BL-6 mice-48 | Effect of bifidobacteria-fermented red ginseng and its constituents ginsenoside rd and protopanaxatriol on anxiety/depression |
| [Wu](https://www.ncbi.nlm.nih.gov/pubmed/?term=Wu Q%5BAuthor%5D&cauthor=true&cauthor_uid=33194428) et al 2020 | - Aβ deposition in cortex and hippocampus - level of insoluble Aβ in the hippocampus and cortex - level of soluble Aβ in cortex but not in hippocampus - microglial activation - IL-1β - TNF-α - IL-4 - IL-6 - INF-γ | -Immunohistochemistry-Immunofluorescence-Thioflavin S staining-Western blot-PCR-ELISA |  | gavage | B. longum 1714 | WT and APP/PS1 mice/- | Effect of **Bifidobacteria** on Aβ accumulation and neuroinflammation |
| [Westfall](https://www.sciencedirect.com/science/article/pii/S0889159120323631?via%3Dihub#!) et al 2021 | - stress-induced IL-1β levels in prefrontal cortex and hippocampus - neuroinflammatory signaling pathways activation | -Open field  -Forced swim  -Bioavailability and pharmacokinetics  -Real time PCR  -ELISA  -Nanostring multiplex assay  -Flow cytometry | 2 weeks | Fed | L. plantarum ATCC 793  B. longum ATCC 15707 | C57BL-6J mice/- | Effect of microbiota metabolites on the T helper 17 to regulatory T cell (Th17/Treg) imbalance promoting resilience to stress-induced anxiety- and depressive-like behaviors |
| Mohammed et al 2019 | - cognitive deficits - hepatic, hippocampal TLR4 expression - BDNF expression and protein - viable cells and shrinking of pyramidal cells in hippocampus | -Novel object recognition task  -MWM  -Biochemical assays  -Real-time PCR  -ELISA | 12 weeks | Gavage | L. Plantarum EMCC-1039 | Wister rats/48 | Effect of probiotics on modulation of hippocampal TLR4/BDNF signal pathway and cognitive impairment |
| [Oh](https://www.ncbi.nlm.nih.gov/pubmed/?term=Oh JH%5BAuthor%5D&cauthor=true&cauthor_uid=32245093) et al 2020 | - spatial memory improvement - BDNF expression - nuclear factor erythroid 2-related factor 2 (Nrf2) phosphorylation | -Western blot and co-immunoprecipitation-Double immunofluorescence staining-MWM | 4 weeks | Gavage | L. plantarum | Sprague Dawley rats/- | Effect of L. plantarum on cognitive improvement |
| [Xie](https://www.ncbi.nlm.nih.gov/pubmed/?term=Xie C%5BAuthor%5D&cauthor=true&cauthor_uid=33120961) and [Prasad](https://www.ncbi.nlm.nih.gov/pubmed/?term=Prasad AA%5BAuthor%5D&cauthor=true&cauthor_uid=33120961) 2020 | - hippocampal dependent cognition - memory impairment induced by the dopamine depletion | -Novel object recognition task-Novel place recognition task-Novelty preference ratio-Elevated plus maze-Immunohistochemistry | 6 weeks | Powdered probiotic was rehydrated with distilled water | L. acticasei  L. rhamnosus | Sprague Dawley rats/31 | Effect of L. acticasei and  L. rhamnosus on hippocampal dependent cognition |
| [Mehrabadi](https://www.ncbi.nlm.nih.gov/pubmed/?term=Mehrabadi S%5BAuthor%5D&cauthor=true&cauthor_uid=32306720) and [Sadr](https://www.ncbi.nlm.nih.gov/pubmed/?term=Sadr SS%5BAuthor%5D&cauthor=true&cauthor_uid=32306720) 2020 | - Aβ plaques - MDA - SOD - IL-1β - TNF-α - inflammation markers | -MWM  -Congo red staining  -Measuring the MDA level and SOD activity  -Assess inflammation markers (IL-1β and TNF-α) of hippocampus  -ELISA | 10 weeks | gavage | L. reuteri  L. rhamnosus  B. infantis | Wistar rats/50 | Effect of probiotics mixture on memory function, inflammation markers and oxidative stress |
| Tahmasebi et al 2020 | - kindling development - escape latency and traveled path in MWM - LTP in hippocampus | -MWM  -LTP | 6 weeks | gavage | L. casei,  L. acidophilus  B. bifidum | Wistar rats/80 | Effect of probiotics and N. sativa extract supplementation on behavioral and electrophysiological effects |
| Shukla et al 2020 | in both cortex and hippocampus   - TLR4 mRNA - IL-1β - IL-6 - IL-10 - TNFα - MCP-1mRNA | -Microbiome analysis  -Blood-brain barrier permeability measurement  -Cytokine mRNA levels  -Plasma cytokine assay  -Immunofluorescence microscopy | 4 weeks  1 day | diet | L. plantarum | Adult C57BL-6 mice/- | Effect of L. plantarum on prevention and mitigation of alcohol-induced neuro-inflammation |
| Toyoda et al 2020 | - gene expression of neurotrophins in hippocampus   preventing psychosocial stress–induced disorders. | -Social interaction test  -Nest building test  -Tail suspension test  -Forced swim test  -Sucrose preference test  -Measurement of gene expression in the hippocampus and neurotransmitter concentration in the brain | 47 days | supplementation | heat-inactivated L. gasseri CP2305 | C57BL6JJmsSlc (B6) mice/- | Effects of dietary intake of heat-inactivated Lactobacillus gasseri CP2305 on stress-induced behavioral and molecular changes in a subchronic and mild social defeat stress mouse model |
